# Modeling of Gap Gene Expression in *Drosophila Kruppel* Mutants

**DOI:** 10.1371/journal.pcbi.1002635

**Published:** 2012-08-23

**Authors:** Konstantin Kozlov, Svetlana Surkova, Ekaterina Myasnikova, John Reinitz, Maria Samsonova

**Affiliations:** 1 Department of Computational Biology/Center for Advanced Studies, St.Petersburg State Polytechnical University, St.Petersburg, Russia; 2 Chicago Center for Systems Biology, Department of Ecology and Evolution, Department of Statistics, and Department of Molecular Genetics and Cell Biology, University of Chicago, Chicago, Illinois, United States of America; University of Wisconsin-Madison, United States of America

## Abstract

The segmentation gene network in *Drosophila* embryo solves the fundamental problem of embryonic patterning: how to establish a periodic pattern of gene expression, which determines both the positions and the identities of body segments. The gap gene network constitutes the first zygotic regulatory tier in this process. Here we have applied the systems-level approach to investigate the regulatory effect of gap gene *Kruppel* (*Kr*) on segmentation gene expression. We acquired a large dataset on the expression of gap genes in *Kr* null mutants and demonstrated that the expression levels of these genes are significantly reduced in the second half of cycle 14A. To explain this novel biological result we applied the gene circuit method which extracts regulatory information from spatial gene expression data. Previous attempts to use this formalism to correctly and quantitatively reproduce gap gene expression in mutants for a trunk gap gene failed, therefore here we constructed a revised model and showed that it correctly reproduces the expression patterns of gap genes in Kr null mutants. We found that the remarkable alteration of gap gene expression patterns in *Kr* mutants can be explained by the dynamic decrease of activating effect of Cad on a target gene and exclusion of *Kr* gene from the complex network of gap gene interactions, that makes it possible for other interactions, in particular, between *hb* and *gt*, to come into effect. The successful modeling of the quantitative aspects of gap gene expression in mutant for the trunk gap gene Kr is a significant achievement of this work. This result also clearly indicates that the oversimplified representation of transcriptional regulation in the previous models is one of the reasons for unsuccessful attempts of mutant simulations.

## Author Summary

Systems biology is aimed to develop an understanding of biological function or process as a system of interacting components. Here we apply the systems-level approach to understand how the blueprints for segments in the fruit fly *Drosophila* embryo arise. We obtain gene expression data and use the gene circuits method which allow us to reconstruct the segment determination process in the computer. To understand the system we need not only to describe it in detail, but also to comprehend what happens when certain stimuli or disruptions occur. Previous attempts to model segmentation gene expression patterns in a mutant for a trunk gap gene were unsuccessful. Here we describe the extension of the model that allows us to solve this problem in the context of *Kruppel* (*Kr*) gene. We show that remarkable alteration of gap gene expression patterns in *Kr* mutants can be explained by dynamic decrease of the activating effect of Cad on a target gene and exclusion of *Kr* from the complex network of gap gene interactions, that makes it possible for other interactions, in particular between *hb* and *gt*, to come into effect.

## Introduction

The segmentation gene network in early *Drosophila* embryo provides a powerful model system to study the role of genes in pattern formation. This network solves the fundamental problem of embryonic patterning: how to establish a periodic pattern of gene expression, which determines both the positions and the identities of body segments [1], [2]. The developmental process which performs this task is called segment determination. The fruit fly segments are arranged sequentially along the anterior-posterior axis of the embryo. All segments are determined simultaneously during the blastoderm stage, just before the onset of gastrulation [3].

The segmentation genes have been subdivided into 4 classes based on their mutant phenotype [1], [2]. The maternal coordinate genes are expressed from the mother and form broad protein gradients in the anterior, posterior or terminal regions of the embryo [4]–[7]. Other genes, which belong to gap, pair-rule and segment-polarity classes, are zygotic, i.e expressed in the embryo. Most of segmentation genes encode transcription factors, which in turn regulate the expression of many other genes, including segmentation genes themselves. It was demonstrated by genetic analysis that segmentation genes form a hierarchical regulatory cascade, in which genes in higher layers (e.g. maternal coordinate genes) regulate genes in lower layers (e.g. gap genes), but not *vice versa*. In addition genes in the same hierarchical level interact with each other.

The gap gene system establishes discrete territories of gene expression based on regulatory input from a long-range protein maternal gradients, Bicoid (Bcd) and Hunchback (Hb) in the anterior and Caudal (Cad) in the posterior of the embryo [8], [9]. Gap genes *Kr*, *kni*, *hb*, *gt* and *tll* are expressed in from one to three domains, each about 10–20 nuclei wide [10]. Early gap gene expression of the trunk gap genes *Kr*, *hb*, *gt* and *kni* is established through feed-forward regulation by maternal gradients, after initial establishment gap domain borders sharpen, moreover both sharpening and maintenance of gap domain boundaries requires gap-gap cross-regulatory interactions [11]. This process is accompanied by the anterior shift of *Kr*, *kni* and *gt* expression domains in the posterior region of the embryo [10], [12], [13].

*Kr* plays a central role in segmental pattern formation as indicated by strong alteration of expression patterns of almost all zygotic segmentation genes in 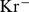 mutants [14]–[16]. *Kr* null mutants show deletion of thoracic and anterior abdominal segments as well as frequent mirror duplications in the abdomen [15], [17]. At the level of gene expression this mutation manifests in the large shift of posterior Gt domain, resulting in overlap of positions of posterior Gt and Kni domains [15], [18]. During sharpening and maintenance stage of gap gene expression Kr acts a repressor of *gt* and *hb* [15], [19], [20]. The repression of *gt*, which expression domains are strictly complementary to those of *Kr*, is strong, while the effect of Kr on *hb* is more subtle [21]–[24]. It was observed in assays with cell lines carrying reporter constructs that the regulatory effect of *Kr* is concentration-dependent: Kr monomer is transcriptional activator, while at high concentrations Kr forms a homodimer and becomes a repressor that function through the same target sequence as the activator. However it is difficult to establish whether such an effect occurs at physiologically relevant regulator's concentrations [25].

The segmentation gene network is one of the few examples of developmental networks studied using data-driven mathematical modeling [13], [26]–[28]. These models fall into two categories. The phenomenological models do not require any *a priory* information about regulatory mechanism [29], [30] and try to reconstruct it by solving the inverse problem of mathematical modelling. A major shortcoming of these models is that their parameters have no explicit connection to the genomic DNA sequence. The second modelling approach seeks to extract information about gene regulation from the sequences of cis-regulatory regions and the measured or inferred binding of sequence-specific transcription factors to these elements [26]–[28], however it still neglects major features of the transcription process, such as chromatin structure and modifications, binding site orientation and proximity to transcription start site, etc. Current simplifications and unknown features limit the predictive power of these models, but more powerful and complex models may be generated in future using better datasets such as *in vivo* transcription factors occupancy, relative accessibility of different DNA regions, *in vivo* data on interplay between different transcription factors, nucleosome and chromatin remodelling enzymes.

In this paper we apply a phenomenological model known as gene circuits to reconstruct the gap gene network in *Kr* null mutants. This model considers a row of nuclei along the A-P axis of the embryo. Between nuclear divisions the model describes three basic processes, namely protein synthesis, protein decay and diffusion of proteins between neighboring nuclei of syncitial blastoderm. A few basic assumptions about eukaryotic transcriptional regulation were incorporated into the model. First a sigmoid regulation-expression function was used to introduce regulatory inputs into the model. Secondly, each regulatory interaction can be represented by a single parameter which sign indicates the type of regulatory interaction: activation (if it is positive), repression (if negative), no interaction (if it is close to zero). Third it was assumed that regulatory inputs are additive and independent of each other.

The gene circuit models were successfully applied to correctly reproduce the quantitative features of gap gene expression in wild type [12], [13]. This study revealed five regulatory mechanisms responsible for sharpening and maintenance of gap gene expression domains: broad activation by maternal gradients of Bcd and Cad; gap gene auto-activation; strong mutual repression between gap genes which show complementary expression patterns (*hb* and *kni*; *Kr* and *gt*); weaker asymmetric repression between overlapping gap genes (Hb on *gt*, Gt on *kni*, Kni on *Kr*, Kr on *hb* and Hb on *Kr*) and repression by terminal gene *tll* at the embryo termini. The asymmetric repression between overlapping gap genes is responsible for shifts of gap gene domains in the posterior region of the embryo. It is important to note that the wild type gap gene circuit model has the predictive power when molecular fluctuations of the input factors are taken into account [31], [32].

It is evident that to understand the gap gene network we need not only to describe the mechanism underlying its functioning in intact state, but also to comprehend what happens when certain stimuli or disruptions occur. Recently Papatsenko and Levine (2011) constructed a dynamic model based on a modular design for the gap gene network, which involves two relatively independent network domains with elements of fractional site occupancy. This model requires only 5–7 parameters to fit quantitative spatial expression data for gap gradients in wild type and explained many expression patterns in segmentation gene mutants obtained in studies published mainly in the late 1980s and early 1990s. However these patterns were characterized qualitatively by visual inspection, that may not capture the fine details of gene expression. For example, previous studies based on qualitative visual analysis of gene expression patterns showed that a *Kr* null mutation results in large shift of posterior Gt domain, overlap of positions of posterior Gt and Kni domains and decrease in the level of *gt* expression in the second half of cycle 14A [15]. Here we obtained a large dataset on gap gene expression in *Kr* null mutants and extracted quantitative gene expression data using a data pipeline established previously [33]. The analysis of this data allowed us to characterize the expression of other gap genes at unprecedented level of detail. In particular we showed that the significant decrease in the level of gene expression in the second half of cycle 14A is common to all gap gene expression domains. This novel biological result seems counterintuitive, because genetics studies show that Kr acts as a repressor, and therefore should come under close scrutiny.

The most serious limitation of the gap gene circuit models is their inability to correctly reproduce the expression patterns in trunk gap gene null mutants at quantitative level, although a theoretical study had shown previously that such prediction is possible if gene circuits models were fit to simulated, noise-free data [29] and simulating null mutants of the terminal gap genes *tll* and *hkb* was successful [12], [13], [34]. A variety of reasons could be responsible for the failure, of which, from our point of view, the most important is the oversimplified representation of transcriptional regulation in the model. Indeed, as was already mentioned above, the action of regulator on its target gene is represented by a single parameter, whereas it is well known that the cis-regulatory elements (CRE) of segmentation genes often reproduce only one of expression domains of an endogenous gene when placed upstream of a reporter gene [35]–[37]. Moreover different CREs of one gene can have different transcription binding site composition, i.e. different regulatory inputs. For example, computational prediction of transcription factor binding sites showed that regulatory sequences which drive expression of *gt* in the anterior and posterior domains have different transcription binding site composition: the anterior *gt* domain has regulatory inputs from Bcd and Kni, while the posterior domain contains inputs from Hb and Cad, which are absent in the sequences responsible for anterior expression [37]. Similar to *gt*, two CREs essential for *hb* expression in anterior domain and in central stripe and posterior domain differ in transcription binding site composition [38]–[40]. It is evident that current gene circuits models do not consider the mechanism of gene regulation at such a level of detail. This defect does not interfere with the ability of these models to fit gap gene expression patterns in wild type, however in mutant background with deficient set of regulators the failure of the model to take into account such features may suddenly become essential.

To avoid such problems we use a revised model which builds on separate treatment of domains with different regulatory inputs. This is possible by narrowing down the spatial domain of the model and considering only the posterior half of the blastoderm (region from 47 to 92% embryo length (EL)), in which each of the trunk gap genes is expressed in one domain.

As opposed to previous gap gene circuit models, which have a constant Bcd gradient and did not consider Cad data from late time points just before the onset of gastrulation [12], [32], and similar to approach used in [30], we implement Bcd as a time-variable input and use data on late Cad expression to represent the rapidly changing expression dynamics of these two genes. After cleavage cycle 12 Bcd nuclear gradient starts to decay [41]. Analysis of data from fixed embryos showed that Bcd protein reached its maximal level near the beginning of cycle 14A and thereafter starts to decrease slowly that culminates in an almost twofold decline by gastrulation [10]. From the second quarter of cleavage cycle 14A onward the *cad* expression in abdominal region start to gradually decrease and by gastrulation *cad* expression in the posterior region sharpens to a stripe which spans from 75 to 90% EL [10].

The gene circuit models do not require any assumption about regulatory interactions within a gene network. Instead the regulatory topology of the network is obtained by solving the inverse problem of mathematical modeling, i.e. by fitting the model to the data [29]. To obtain the estimates for regulatory parameters that predict a specific network topology in mutants we fitted the model to gap gene expression patterns in wild type and in embryos with homozygous null mutation in *Kr* gene simultaneously. The logical justification of such an approach is to use the parameters of the wild type gap gene network as specific constraints on regulatory weights in mutants in order to obtain the consistent parameter estimates for both genotypes on one hand and on the other hand to preserve the characteristic features of gene regulation in mutant. The parameter estimates obtained in such a way were further studied by applying identifiability analysis, that confirmed that fitting to two genotypes simultaneously substantially increases the statistical significance of parameter values.

We use the modeling framework outlined above to explain the characteristic features of gap gene expression in *Kr* null mutants and in the posterior half of the blastoderm. In what follows we describe the expression patterns of gap genes in *Kr* null mutants and analyze quantitative gene expression data extracted from these patterns. We then use these data as input to a new gap gene circuit model. We show that in contrast to earlier models, this model correctly reproduces the characteristic features of gap gene expression in *Kr* mutants. In particular, it reproduces correctly the greater shift of posterior Gt domain than in wild type and significant decrease in the level of gap gene expression in the second half of cycle 14A. We next obtain the parameter estimates for the model (and hence the predicted gap gene network topology in wild type and mutant) and perform identifiability analysis to understand how reliable are these estimates. We study the dynamical behavior of our model and analyze the role of individual regulatory loops in gap gene expression in wild type and mutants. We show that a remarkable transformation of gap gene expression patterns in *Kr* mutants can be explained by dynamic decrease of activating effect of Cad on a target gene and exclusion of *Kr* gene from the complex network of gap gene interactions, that makes it possible for other interactions, in particular, between *hb* and *gt*, to come into effect. Our model also predicts the derepression of the anterior border of Hb posterior domain in *Kr*;*kni* double mutants, that is established in the absence of key repressors. We validate this prediction and show the correctness of network topology inferred in this work.

## Results

### Gap gene expression in *Kr* mutants

**Figure 1:**
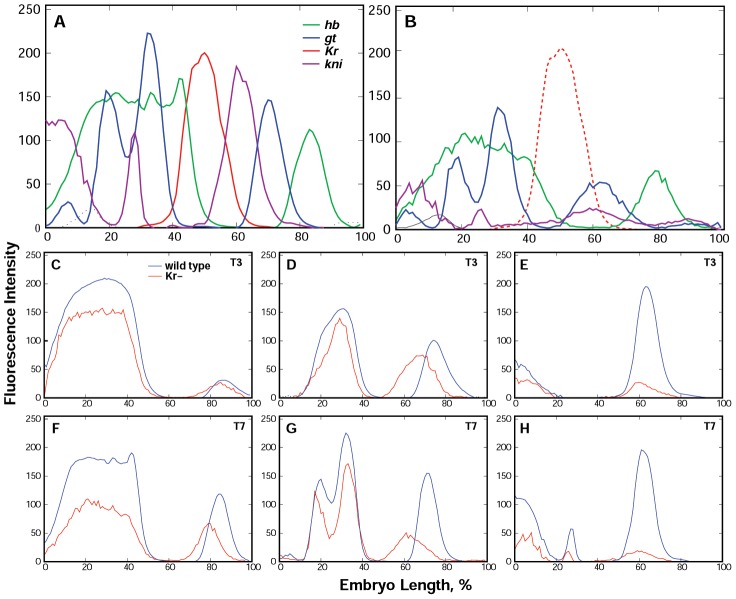
The main features of gap gene expression in *Kr* mutants as compared to wild type embryos. A,B. Integrated patterns of gap gene expression in wild type embryos (A) and in *Kr* mutants (B). The red dotted line in (B) shows *Kr* expression in wild type embryos. C–E. *hb*, *gt* and *kni* expression in *Kr* mutants and wild type embryos from time class 3. F–H. Expression of the same genes in time class 7.

In wild type *Drosophila* embryos gap genes are expressed as large intersecting domains along the A-P axis (Figure 1A). In general, all these domains exhibit similar temporal dynamics: after formation they start to grow, reach maximum expression levels around mid-cycle 14A and decline by gastrulation. In the course of cycle 14A gap gene domains change their positions and shift to the anterior [10]. We have shown that the asymmetric gap-gap cross-repression with the posterior dominance is responsible for these shifts [12].

In *Kr* null mutants gap gene expression is significantly altered (Figure 1B). It has been previously reported that in these mutants the posterior domain of *gt* is expanded towards the center of the embryo [42], [43]. We detect that in the course of cycle 14A the posterior domain of *gt* shifts dynamically on 15% embryo length (EL) in the anterior direction and overlaps with Kni domain. Thus, by gastrulation, the difference in position of *gt* domain in mutants and wild type embryos constitutes approximately 10% EL. The anterior shift of the Kni domain maxima in *Kr* mutants constitutes only 1.8% EL. Hb posterior domain in mutants is formed at the beginning of cycle 14A and shifts on about 3% EL in the anterior direction during this cycle. Thus, the positional dynamics of this domain in mutants and wild type is similar.

The level of *hb* posterior expression in mutants is nearly the same as in wild type until time class 3, but declines afterwards. By gastrulation it constitutes only a half of the wild type expression level (Figure 1C,F). Gt posterior domain is initially lower than in wild type, it grows up to time class 4 and significantly declines thereafter (Figure 1D,G). The level of *kni* expression remains constantly low throughout cycle 14A (Figure 1E,H) with a slight decrease at the very end of this cycle (not shown).

The features of the gap gene expression in *Kr* mutants described above raise many questions. Namely, the regulatory mechanisms underlying a decrease in gene expression levels, as well as a much larger shift of Gt posterior domain should be explained. In the following sections, we describe our modification of the gene circuit model [12], [13], [29], [44] that correctly reproduces the gap gene expression in *Kr* mutants and hence can serve as a tool to answer these questions.

### Model fitting

The gene circuit model used in this work differs from previous implementations in several aspects. First we narrowed down the spatial domain of the model by considering only the posterior half of the blastoderm (region from 47 to 92% embryo length (EL)), in which each of the trunk gap genes is expressed in one domain. This allows us to avoid the inherent limitation of the model, in which the action of regulator on its target gene is represented by a single parameter.

Secondly, as opposed to previous gap gene circuit models which have a constant Bcd gradient and did not consider Cad data from late time points just before the onset of gastrulation [12], [32], we implement Bcd as a time-variable input and use data on late Cad expression to represent the rapidly changing expression dynamics of these two genes at that stage. We used *bcd* and *cad* profiles from FlyEx database for cycle 13 and eight temporal classes of cycle 14A as external inputs to our model equations.

We used the modeling framework outlined above to explain the characteristic features of gap gene expression in *Kr* null mutants and in the posterior half of the blastoderm. To obtain the estimates for regulatory parameters that predict a specific network topology in mutants the model was fitted to gap gene expression patterns in wild type and in embryos with homozygous null mutation in *Kr* gene simultaneously. DEEP method was applied to minimize the sum of squared differences between experimental observations and model patterns [45], [46] and find all parameters of model equations, i.e. regulatory weights, synthesis rates, decay and diffusion constants, that allow to reproduce the characteristic features of gap gene expression in *Kr* null mutants as closely as possible. We performed over 200 runs with different initial parameter approximations and control variables. The search space was sampled uniformly for each parameter in the interval defined by biologically relevant limits. Two step procedure was applied to construct the ensemble of parameter sets. On the first stage, the residual mean square (*RMS*) was checked and the sets with *RMS* less than 5% of the maximal gene expression value (equals 255 in our data) were accepted for further analysis. Secondly, we inspected the model expression patterns visually. Consequently, the ensemble of 11 parameter sets was obtained that correctly reproduces the dynamics of gene expression in wild type and mutant embryos, in particular the decrease of gap gene expression levels and the anterior shift of *gt* domain.

### Gene network topology

**Figure 2:**
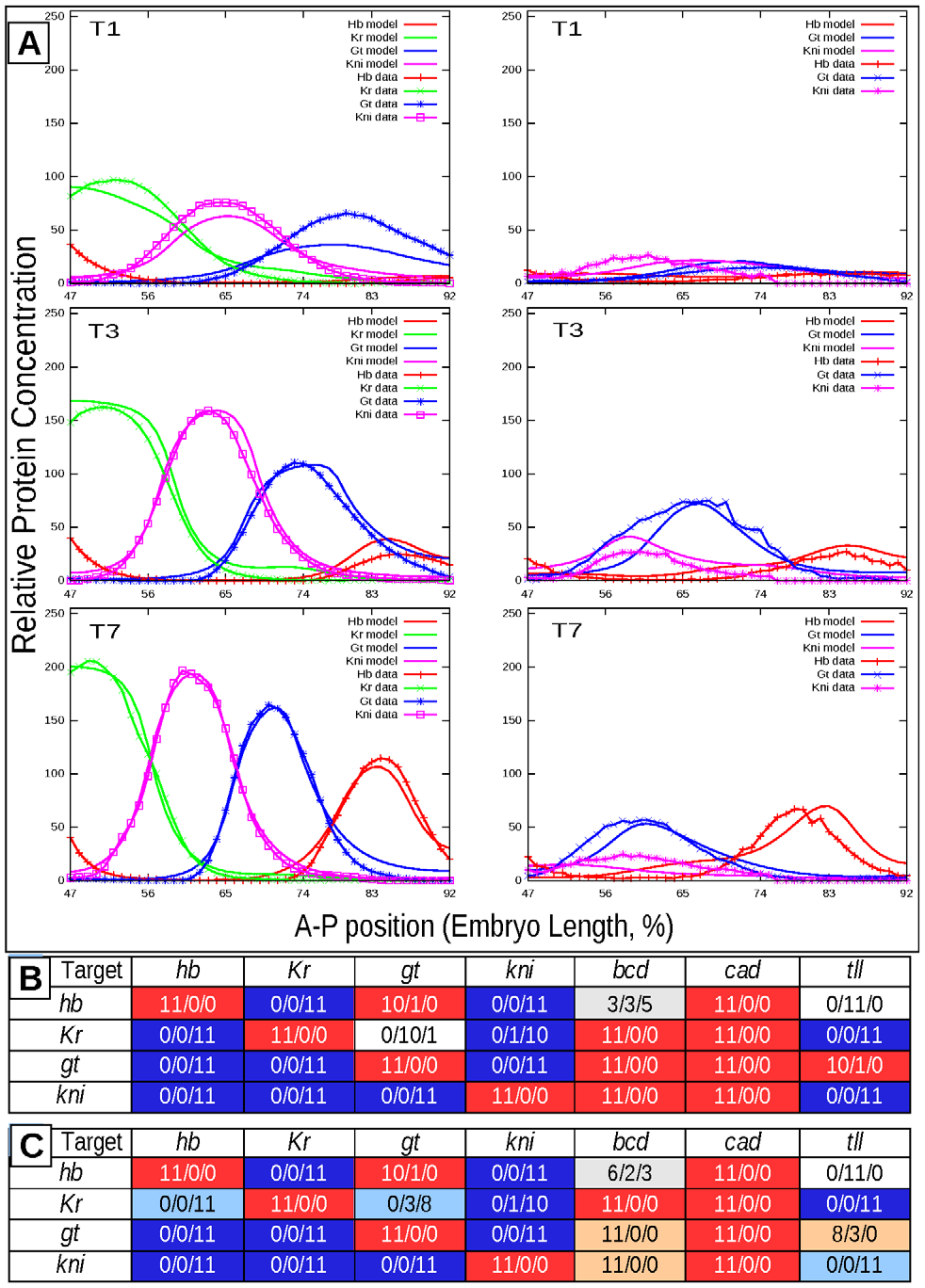
Model output for a representative consensus network compared to quantitative gene expression data and predicted regulatory network topology. A. Right panels display simulation results and data for mutant genotype, left panels show simulation results and data for wild type. Top, middle and bottom panels present data and profiles for first, third and seventh temporal classes, respectively. Wild type data was taken from the FlyEx database [75], [84]. B. Prediction of network topology based on classification of regulatory weights. C. Prediction based on both classification and identifiability analysis of regulatory parameters. Rows correspond to target genes; columns correspond to regulators. Numbers in cell define in how many circuits a given interaction was classified as activation, no action or repression. Red, blue and white cells mark activation, repression and no action correspondingly. Light red and light blue cells define insignificant activation and insignificant repression correspondingly. Cells colored in grey define the situation in which a parameter takes both negative and positive values and therefore the type of regulation is not obvious.

To infer the topology of regulatory network we classified the estimates of regulatory weights (the elements of *T* and *E* matrices) into the following three categories: ‘activation’ (parameter values greater than 0.005), ‘repression’ (parameter values less than −0.005) and ‘no interaction’ (between −0.005 and 0.005). This leads to a predicted regulatory topology of the network based on which category a majority of parameter estimates falls into (summarized in Figure 2).

There are several networks in the ensemble called consensus networks, in which the signs of regulatory parameters coincide with the predicted network topology inferred from the fits. Figure 2A shows simulation results together with experimental data and Table S1 presents parameters for one of such networks. It is evident that in spite of some patterning defects especially at early stages the model correctly reproduces the dynamics of gene expression in wild type and mutant embryos.

Some basic features of the gap gene network topology in wild type and mutant become immediately obvious from inspection of Figure 2B and Table S1. First, Cad activates zygotic gap gene expression. Second, *hb*, *Kr*, *kni*, and *gt* show autoactivation. Third, Bcd activates *Kr*, *gt*, *kni* in all parameter sets, however in case of *hb* it shows activation in approximately the same number of circuits as it shows repression. Fourth, all reciprocal interactions among trunk gap genes are either zero or repressive. An important exception is activation of *hb* by Gt. Finally *tll* represses *kni* and *Kr* and weakly activates *gt*.

### Parameter identifiability and correlations

The identifiability analysis was conducted with respect to parameters of the model estimated by fitting to experimental data. The model considers the time evolution of protein concentrations of four gap genes *hb*, *Kr*, *gt*, and *kni* in two genotypes: wild type and in embryos with homozygous null mutation in *Kr* gene. The total parameter set that minimizes the cost functional 0 consists of 40 parameters and is denoted as 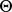. The set includes four subsets 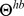, 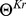, 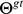, and 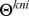 of 10 parameters each, that describe regulatory action on each target gene. In mutants the model is only fitted to quantitative gene expression data for 3 genes, *gt*, *hb* and *kni*, and hence the parameters from 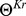 are estimated using data points from wild type embryos only (half of all data points). All the other parameter subsets are estimated from the whole dataset. Due to lack of space we denote the elements of inter-connectivity matrices *T* and *E* by single-letter notations of genes, namely, *H*, *K*, *G*, *N*, *B*, *C*, and *T* stand for *gt*, *Kr*, *gt*, *kni*, *bcd*, *cad*, and *tll*, respectively. For example, 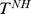 characterizes the regulatory action of Hb on *kni*.

The sensitivity of the model solution to parameter changes is characterized by the size of confidence intervals. The confidence intervals (2) (see Methods) are constructed under the assumption of normally distributed error in data, that is not satisfied for gene expression data. The error in data almost linearly increases with the mean concentration that is typical rather for the Poisson than for normal distribution. To make the error independent of the mean we applied the variance-stabilizing transform 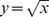 to both data and model solution. The transformed objective functional was minimized using the parameter estimates obtained for non-transformed functional as initial values for the optimization procedure. The new solutions were found in a very close vicinity of initial parameter sets.

The 11 parameter sets, which minimize the transformed model functional, are given in Table S2 and will be referred to as circuit parameter sets. The newly estimated regulatory weights were classified into regulatory categories as described in subsection Gene Network Topology. This classification results in predicted regulatory topology of the network (Figure 2C), which is largely the same as in Figure 2B, however not all the entries in two tables coincide. The estimates of some parameters not-uniquely determine the type of gene regulation in different circuits, i.e. in some circuits the parameter estimates exceed the threshold 0.005 in absolute value, while in the others are below the threshold. It is also true for new parameter sets, however, the number of such circuits is different than those given in Figure 2B.

**Figure 3:**
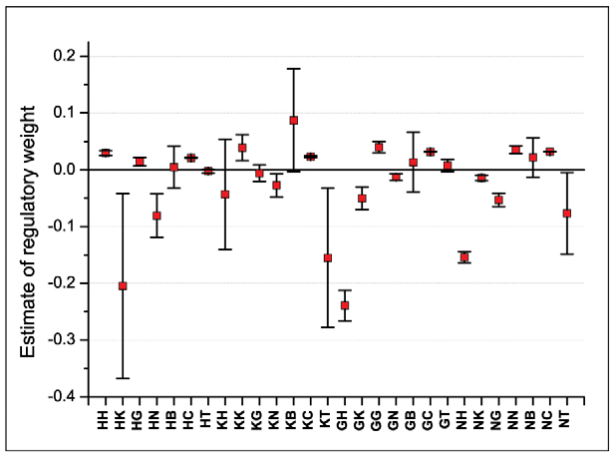
95% confidence intervals for estimates of parameters of a consensus circuit (circuit C5, see Table S2). Regulatory weights are labeled by single-letter notations of genes: *hb*(H), *Kr*(K), *gt*(G), *kni*(N), *bcd*(B), *cad*(C), *tll*(T). The first letter corresponds to the target gene (e.g., HK stands for 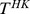).

The confidence intervals for individual parameters are constructed in the vicinity of the model solution. The results for one representative circuit are presented in Figure 3. Most of the values of regulatory parameters are very close to zero, and it is important to make sure whether the value (more precisely, the sign) of a regulatory parameter is significant. The hypothesis that the parameter estimate is non-zero is tested as follows: if a confidence interval includes both positive and negative values, the hypothesis is rejected, otherwise, accepted.

Our classification method to infer the topology of regulatory network used in this work, was based on comparison of the values of regulatory parameters with the threshold 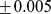. However, as it has already been mentioned, estimates of some parameters take values, which only exceed the threshold in part of circuits. By exploration of confidence intervals for these parameters we came to the conclusion, that almost all the estimates, that are close in absolute value to the threshold, are insignificant. This result explains the discrepancies between the network topologies presented in Figures 2B and 2C: the conclusions about the type of gene interaction that are based on insignificant parameter estimates are unreliable.

The analysis of confidence intervals conducted for all the circuits (Figures S2 and S3) allowed us to refine the predicted regulatory network topology (Figure 2C). We classify parameters as insignificant activation/repression if the parameter estimates are positive/negative in almost all the circuits but their confidence intervals contain zero, and hence the parameter sign cannot be identified. As a result the non-identifiable regulatory parameters are 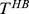, 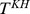, 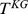, 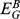, 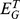, 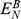, and 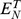 and therefore we cannot draw any conclusion about these interactions. Interestingly most of these interactions involve *Kr* as a target gene or Bcd as a regulator of gap gene domains located in the posterior of the embryo. Other regulatory parameters are well identifiable and, hence, the identifiability analysis corroborates the gene network topology drawn from classifying parameter values only.

It should be stressed that the confidence intervals provide the full information about the parameter estimates only in case of parameter independency, otherwise the intervals are overestimated. Moreover, strong correlation between parameters may lead to their non-identifiability, because a change in one parameter value can be compensated by the appropriate changes of another parameters and, hence, does not significantly influence the solution. In view of this we investigate the dependencies between parameters using the collinearity analysis of the sensitivity matrix. This method allows to reveal correlated and hence non-identifiable subsets of parameters.

The sensitivity matrix defined in Methods was analyzed in the vicinity of 11 points in the parameter space that define the optimal model solutions. The collinearity index 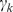 (equation (3) in Methods) was computed for all the subsets of dimension *k* of the parameter set 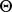. The threshold value for 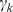 was chosen equal to 7. This value in case of 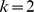 corresponded to approximately 99% pairwise Pearson correlation between columns of the sensitivity matrix. The method allowed to detect subsets of dimension 2 and 3 with the collinearity index exceeding the threshold value, i.e. subsets of poorly or non-identifiable parameters.

Most of parameter combinations in these subsets were the same for all 11 circuits (see Table 1). Almost all the pairs of parameters in subsets of dimension 2 belonged to 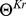, i.e. were related to *Kr* target gene. To explain this result we additionally compute the collinearity indices 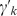 between columns of the upper half of the sensitivity matrix, that only include the partial derivatives computed at 1532 wildtype observations. The method detected much more parameter subsets with collinearity indices 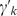 greater than the threshold, that included the parameters characterizing the input of all four genes. The parameters of the full model fitted to two genotypes thus are better identifiable than those of the model that solely describes the wild type data. However, the parameters from 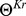 cannot be identified from the full model as are just estimated from the wild type observations.

**Table 1:** Two- and three-dimensional subsets of regulatory weights with mean collinearity indices higher than 7.

Parameter combinations			Collinearity index	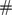
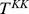	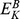		12.21	10
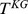	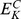		10.27	1*
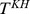	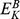		9.99	11
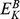	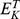		9.43	11
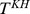	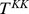		7.93	7
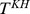	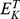		7.76	11
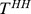	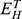		7.16	5
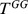	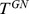	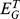	10.03	8
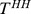	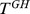	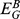	7.42	4
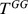	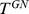	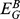	12.11	1*
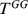	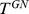	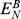	8.27	1*
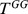	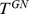	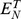	10.26	1*
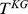	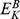	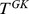	9.75	1*
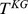	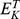	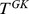	9.54	1*
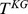	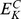	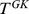	8.76	1*

Column 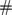 is the number of circuits in which the collinearity index exceeds the threshold value. The parameter combinations marked by * were only detected in a single C6 circuit (see Table S2). Regulatory weights are labeled by single-letter notations of genes: *hb*(H), *Kr*(K), *gt*(G), *kni*(N), *bcd*(B), *cad*(C), *tll*(T).

The subsets of dimension 3 with the highest collinearity indices (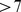) are also common for the most of the circuits. Most of these combinations are related to *gt* and *Kr*, i.e., include parameters from 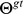 and 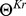.

The two approaches applied to characterize parameter identifiability are closely connected and complement each other. By exploration of confidence intervals we can see to what extent the model solution is sensitive to parameter changes and test the significance of parameter sign, but this method does not give any explanations to the sources of non-identifiabilities. One of such explanations can be provided by collinearity analysis. The correlation between parameters revealed by this approach can clarify insignificance or unreliability of parameter estimates with large confidence intervals. For example, we derive non-identifiability of 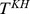 from the large size of its confidence interval and at the same time the analysis of the sensitivity matrix allows us to detect the subset of parameters 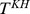 and 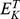 with high mean collinearity index equal to 7.76 (see Table 1). Thus, poor identifiability of 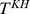 can be explained by correlation between two regulatory parameters, that is reflected in their high collinearity index.

The gene network topology inferred from both classifying the parameter values and parameter identifiability analysis is presented in Figure 2C. As Figure S4 shows it is largely in agreement with topologies predicted by earlier models [13], [31], [34], [47]. Strong constraints for mutual repression are present for *kni* and *hb*, which show complementary expression patterns. Besides, strong repressive action exert both Kr on *hb* and Hb on *gt*. Some previous models had predicted the repressive action of Kr on *hb* [31], while most showed no interaction [13], [34], [47]. Many repressive interactions between gap genes show weaker constraints toward repression, and interestingly we have found very weak or no dynamical constraints for repression of Gt on *Kr*, the interaction with strong constraint for repression in all wild type gene circuit models [13], [31], [34], [47], [48]. In addition our model predicts weak repressive interactions between Kni and *gt* and Kr and *kni*. In earlier gap gene circuit models the first interaction was predicted as no interaction [13], activation [34] or activation in about half the circuits, and repression in the other half [31]. The repressive action of Kr on *kni* is only observed in our model, all other models predicted no interaction between the two genes. In addition in current model Bcd shows activation of *hb* in approximately the same number of circuits as it shows repression, while in all previous models this interaction was predicted as activation. Weak activation of *gt* by Tll is now present in 10 parameter sets, while previous results predicted this interaction as repression. Finally our model predicts no interaction between Tll and *hb*. Some previous models had classified this interaction as activation [31], [34], while other predicted it as repression or no interaction [13], [48], [49].

### Mechanism of alteration of gap gene expression patterns in Kr mutants

Null mutation in *Kr* gene results in strong alteration of expression patterns of almost all zygotic segmentation genes. In gap gene network this mutation manifests in significant reduction of gap gene expression levels in cycle 14A, as well as in large shift of posterior Gt domain and overlap of positions of posterior Gt and Kni domains.

Previous gap gene circuit models fail to correctly model the gap gene expression patterns in the embryos homozygous for null mutation in a trunk gap gene. A new model introduced here correctly reproduces the characteristic features of gap gene expression in *Kr* null mutants and in the posterior half of the blastoderm. To investigate the mechanism responsible for strong alteration of the expression patterns of gap genes in *Kr* null mutants we have performed the detailed graphical analysis of gap gene regulation in the posterior of the embryo. This analysis revealed the following regulatory principles.

In both *Kr* mutants and wild type the posterior Hb domain is the last gap domain to form; its expression is initiated in cleavage cycle 13 and the domain retracts from the posterior pole at temporal class 2 of cycle 14A. Later the expression level in the posterior Hb domain increases gradually up to temporal class 7 and diminishes at the very end of cycle 14A in wild type embryos, while in mutants the level of expression is nearly the same as in wild type until time class 3, but declines afterwards.

In the model, combined activating inputs by Cad and Gt are responsible for *hb* expression in early cycle 14A. At later time (from time class 3 onward) *hb* autoactivation starts to play role and it gradually supplements activation by other factors, which strength decreases in both genotypes. In mutants the positional dynamics of Hb domain resembles that in wild type, while the anterior shift of Gt domain is much larger. Larger anterior shift of Gt domain causes stronger decrease in activation contribution by *gt* to the Hb domain (Figure S5). This effect together with smaller autoactivation level may cause the fall in accumulation of Hb in *Kr* mutants. We do not include *hkb*, the gene responsible for formation of the posterior boundary of the Hb posterior domain, in our model and therefore only the mechanism underlying the formation of the anterior boundary can be analyzed. In wild type this boundary is formed by joint repression by Kr and Kni, while in *Kr* mutants Kni is the only repressive input, which strength diminishes with time due to decrease in *kni* expression level.

Gt posterior domain forms in cycle 13. In wild type embryos the expression of *gt* in this domain reaches maximum at time class 5 and then declines. In *Kr* null mutants *gt* expression is lower than in wild type, it grows up to time class 4 and significantly declines thereafter (Figure 1D,G). In mutants the anterior shift of *gt* posterior domain is much larger than in wild type: by gastrulation, the difference in position of *gt* domain in mutants and wild type embryos constitutes 10% EL and Gt domain overlaps Kni domain.

**Figure 4:**
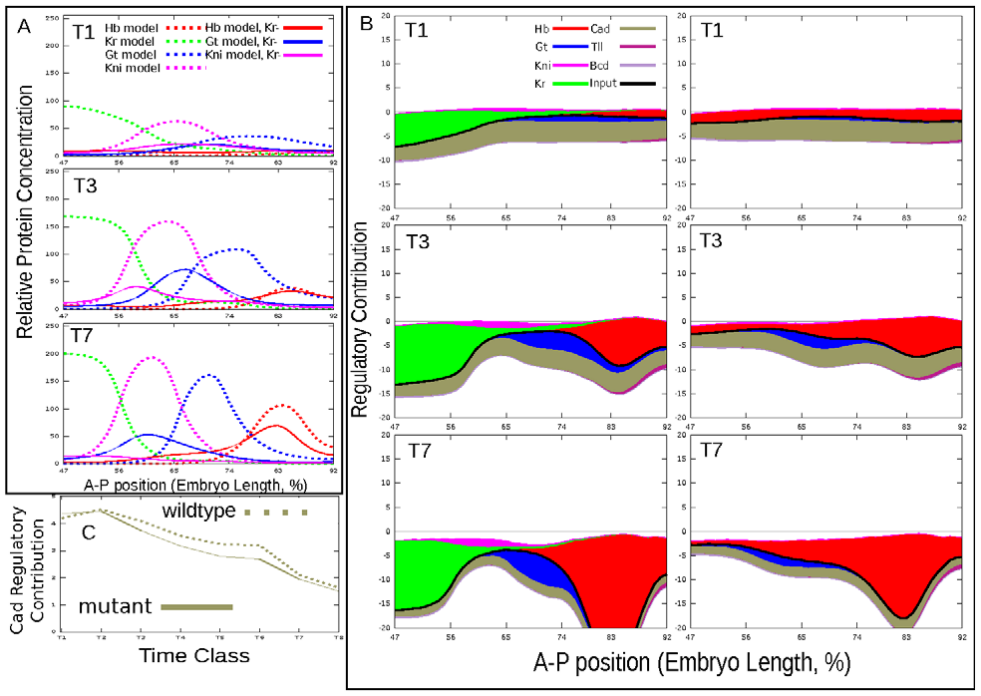
Interactions involved in regulation of Gt posterior domain. A. Modeled expression patterns at temporal classes T1, T3 and T7. B. Regulatory contributions in wild type (center) and mutant (right). C. Temporal change in regulatory contributions at position corresponding to Gt domain maximum. Colored areas are given by 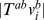 or 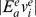 in equation (1) and reflect the strength of a given interaction at a specific point in space and time. All plots are based on best scoring solution (circuit C9, see Table S1 for parameters).

Cad and Gt autoactivation contributes activating inputs on posterior Gt domain (Figure 4). Both in *Kr* mutants and wild type embryos the strength of Cad activating input decreases by gastrulation, however in mutants this reduction is larger, as Gt domain shifts closer to the anterior end of the embryo against gradient of Cad concentration (Figure 4C). It is obvious that weaker activation of *gt* by Cad will lead to lower level of *gt* autoactivation within its domain. Indeed, by time class 7 autoactivation contributes strongly to expression of *gt* only in wild type. This provides a straightforward mechanism for reduction in the level of *gt* expression in the posterior of the *Kr* mutant embryos: the decrease of activating contribution by Cad and diminishing *gt* autoactivation result in downregulation of *gt* expression.

It should be noted that in mutant a small level of Hb repression is evident across the middle region of the model spatial domain at all times (Figure 4B). This repression is caused by the spurious expression of *hb* in the region of 60–77%EL (see Figure 1) and could be responsible for decrease in the *gt* expression level. However the exclusion of this elevated expression from the model (by setting Gt input into *hb* expression to zero) does not lead to increase in the *gt* expression level (Figure S6) in mutants, that makes it unlikely that Hb repression contributes significantly to the low levels of *gt*.

Kr and Kni repression is involved in the positioning of the anterior boundary of the Gt posterior domain in wild type embryos (Figure 4). In mutants Kni does not significantly contribute to this boundary formation, that can be accounted for its small expression level. Besides by time class 7 Gt shifts to the anterior border of the model spatial domain. All this precludes the conclusions on the possible mechanisms of the anterior boundary formation of Gt domain in *Kr* mutants.

The posterior boundary of the Gt domain depends almost exclusively on very strong repression by Hb both in wild type embryos and *Kr* mutants (Figure 4). The accumulation of Hb in the posterior region causes increase in both levels and extent of this repression over time. This in turn leads to an anterior shift of Gt domain. In *Kr* null mutants the lack of *gt* repression by Kr and very weak repression of *gt* by Kni allows Gt posterior domain to move further than in wild type to the territory of *kni* expression. Thus, the mechanism underlying the shift in posterior Gt domain in *Kr* mutants is equivalent to those of other gap domains in wild type embryos: shift happens because of the almost absence of repression by the adjacent anterior domain (Kni), while it becomes increasingly repressed posteriorly (by Hb, in this case).

In wild type embryos *kni* expression is first detected in cycle 13; it reaches maximum by temporal class 5 of cycle 14A. In *Kr* mutants the level of *kni* expression remains constantly low throughout cycle 14A (Figure 1E,H) and the anterior shift of the Kni domain maximum constitutes only 1.8% EL.

**Figure 5:**
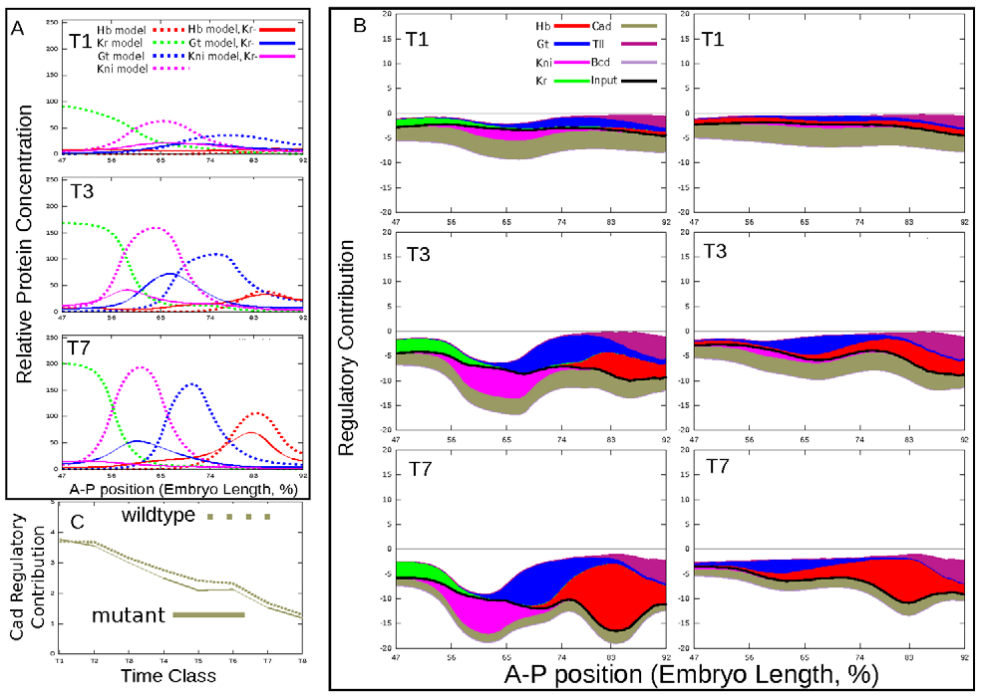
Interactions involved in regulation of Kni posterior domain. A. Modeled expression patterns at temporal classes T1, T3 and T7. B. Regulatory contributions in wild type (center) and mutant (right). C. Temporal change in regulatory contributions at position corresponding to Kni domain maximum. Colored areas are given by 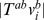 or 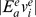 in equation (1) and reflect the strength of a given interaction at a specific point in space and time. All plots are based on best scoring solution (circuit C9, see Table S1 for parameters).

In the model *cad* and *kni* autoactivation provides activating inputs on Kni domain. Both in *Kr* mutants and wild type embryos the strength of Cad input decreases by gastrulation, however in mutants this decrease is stronger, happens faster and is accompanied by diminishing *kni* autoactivation. By temporal class 7 autoactivation of *kni* is present at significant level only in wild type. Similar to Gt domain a small level of *hb* repression evident across the middle region of the model spatial domain (Figure 5B) is unlikely to contribute to the low levels of kni as the exclusion of this spurious expression from the model does not lead to increase in the *kni* expression level (Figure S6).

In wild type embryos Kr repression is responsible for positioning the anterior boundary of Kni domain, while in *Kr* mutants this boundary forms outside the model spatial domain. The posterior boundary of this domain depends on repression by Gt, Hb and Tll in both genotypes, however Tll repression is only retained in a region posterior of 80% EL. In *Kr* mutants Gt repression spreads into a territory where *kni* expression domain forms, preventing the increase of gene expression level in this domain.

**Figure 6:**
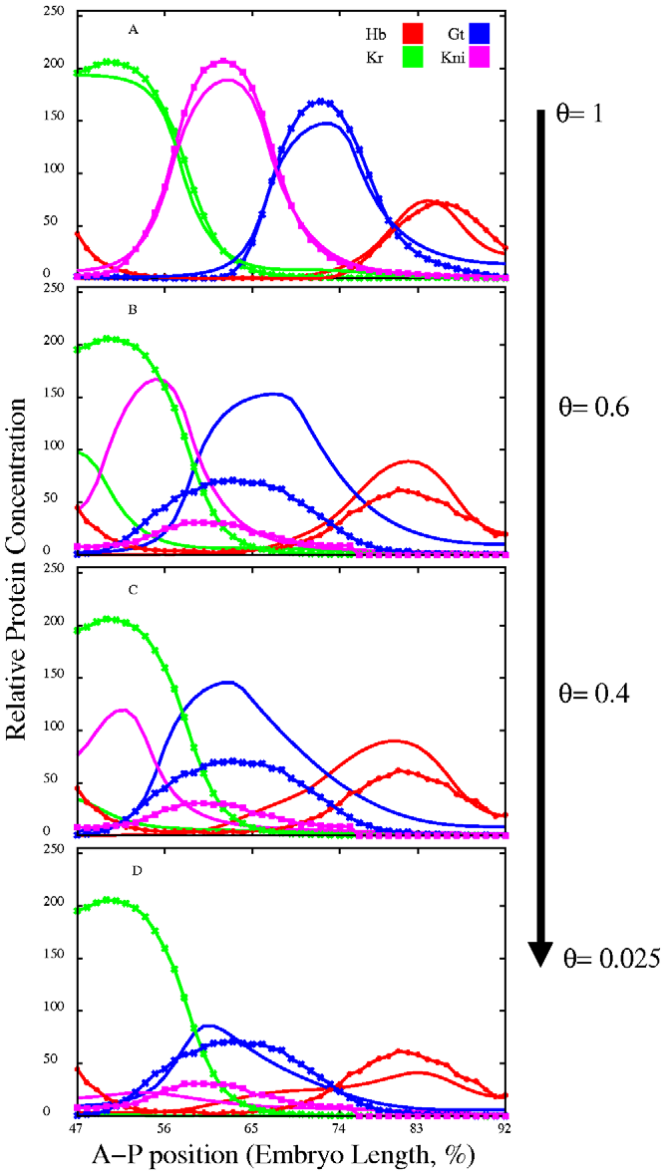
Simulation of *Kr* null mutants. A. Modeled expression patterns (lines) and experimental data (lines with symbols) on gap gene expression in wild type. B–D. Data on gap gene expression in *Kr* mutant (lines with symbols) and model output (lines) at the values of 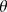 equal to 0.6, 0.4 and 0.025. 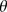 decrease is indicated by arrow.

The analysis performed above points on the central role of *hb* in gap gene regulation in 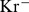 embryos. To support the validity of this prediction the *in silico* experiments were done. In these experiments, instead of setting 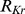 to zero (as is usually done to model mutant genotype), we multiplied it to the scaling coefficient 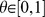, which gradually decreases from 1 to 0, and inspected changes in gap gene expression in the posterior of the embryo. It is evident in Figure 6 that decrease in Kr regulatory input is most important for *hb* dynamics. Besides, this experiment demonstrates the monotonous dependence of change of gap protein concentrations on 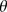, that may be an additional argument for validity of numerical results, obtained independently in two genotype modeling. It should be stressed that though such an experiment does not has a special biological sense, it makes it possible to predict, which component of the network is subject to biggest impact by mutation. The last hardly could be revealed in biological experiments.

## Discussion

In this paper we investigate the mechanism of alteration of gap gene expression patterns in null mutants for one of trunk genes, *Kr*. We applied the integrative approach, which combines on one hand the characterization of expression of gap genes in *Kr* mutants in a quantitative manner and on the other the gene circuit method, a system-level approach to refine gap gene network topology and reveal mechanisms responsible for the alteration. However before considering these biological questions in detail below, we wish to discuss several improvements in gene circuits method, which for the first time make it possible to correctly reproduce gap gene expression patterns in mutant for a trunk gap gene. We believe that these methodological improvements have important implications for reverse engineering of gene networks in general.

### Revised gene circuit model

The failure of gene circuit models to correctly reproduce expression patterns in gap gene null mutants could be due to a variety of reasons: it is possible that our data does not correctly reflect the absolute concentrations of gap gene proteins and some scaling of expression patterns in the data is necessary or production delays may be required in the model to eliminate premature initiation of gap-gap gene interactions. Alternatively, a more detailed consideration of molecular mechanisms responsible for gap gene expression may be required for mutant simulation. At present we do not have a satisfactory understanding of such mechanisms for any of the gap genes, however some important regulatory principles emerged from experiments with reporter constructs, DNAse protection assays, Chip-chip experiments and large-scale computational screens to identify and analyze gap gene enhancers. These experiments demonstrated that cis-regulatory elements (CRE) of segmentation genes often reproduce only one element of an endogenous gene expression pattern when placed upstream of a reporter gene [35]–[37] and that different CRE of one gene can have different transcription binding site composition, i.e. different regulatory inputs. For example, three *gt* CREs drive reporter gene expression in the posterior (*gt_(-3)*) and distinct anterior domains (*gt_(-6)*, *gt_(-10)*), respectively, while another element (*gt_(-1)*) reproduces endogenous *gt* expression in both anterior and posterior domains [36], [37]. Moreover computational screens predict that the anterior and posterior *gt* domains have different regulatory inputs. It is currently unclear how *gt* CREs interact in regulation of the endogenous *gt* gene, however it is obvious that the representation of the gap gene regulatory interactions by a single parameter in the gene circuit model is hardly suitable for theoretical description of such a complex mechanism and should be substituted by more realistic representation.

As a first step in this direction we introduced a revised model which builds on gene circuit method but treats domains with different regulatory inputs separately. A straightforward way to implement such a modification is to narrow down the spatial domain of the model by considering only the posterior half of the blastoderm, in which each of the trunk gap genes is expressed in one domain. Here we demonstrated that the new model correctly reproduces the characteristic features of gap gene expression in *Kr* mutants, the greater shift of posterior Gt domain than in wild type and significant decrease in the level of gap gene expression in the second half of cycle 14A in particular. The successful modeling of expression patterns in a mutant for a trunk gap gene is a significant achievement of this work. This result also clearly indicates that the oversimplified representation of transcriptional regulation in the previous models is one of the reasons for unsuccessful attempts of mutant simulations.

Most of previous gap gene circuit models represent Bcd as a time-constant gradient and did not consider Cad data from late time points just before the onset of gastrulation [12], [32]. It was reasonable to implement such an approach when the mechanism for precise positioning of segmentation gene expression domains was investigated, however an intriguing feature of *Kr* mutants is the reduction in the level of gap gene expression in the second half of cycle 14A, a phenomenon which understanding requires a precise consideration of activators responsible for gap gene expression. As we have shown before [10], Bcd protein reaches its maximal level near the beginning of cycle 14A and thereafter starts to decline slowly, while Cad expression in abdominal region starts to gradually decrease from time class 3 onward. Accordingly in the model we implement Bcd as a time-variable input and use data on late Cad expression to represent the rapidly changing expression dynamics of these two genes. This allows us to demonstrate that decrease of activating input by Cad and weakening of autoactivation are responsible for reduction in the level of *gt* and *kni* expression in the posterior of the *Kr* mutant embryo.

In gene circuit models the regulatory topology of the network is obtained by solving the inverse problem of mathematical modeling, i.e. by fitting the model to the data [29]. To obtain the estimates for regulatory parameters that predict a specific network topology in mutants we fitted the model to gap gene expression patterns in wild type and in embryos with homozygous null mutation in *Kr* gene simultaneously. The rationale behind such an approach is that, as it was shown in the Parameter identifiability and Correlations section, using the parameters of the wild type gap gene network as specific constraints on regulatory weights in mutants substantially increases the statistical significance of fitted parameter values.

Our results demonstrate the existence of parameter sets describing gap gene expression in two genotypes simultaneously and thus the applicability of the gap gene circuit formalism to model genotypes of trunk gap gene mutants. One finds hard to say whether the overfitting is the reason why these parameter sets were not discovered during the fit to the wild type data alone. Overfitting is defined by a fine balance between the number of model parameters and the level of details used to describe the system. The qualitative models usually require small amount of parameters. However, when the data under modelling becomes more quantitative, the number of parameters usually increases, and in the general case there are no methods to find the optimal number of parameters, exceeding which will lead to overfitting.

We treat the possible overfitting problem by applying the practical identifiability analysis of the found parameter values. The parameter estimates obtained in such a way were further studied by applying identifiability analysis. Two approaches were used. First the sensitivity of the model to parameter changes and identifiability of parameters in the vicinity of the model solution were analyzed on the basis of confidence intervals of parameter estimates. This analysis showed that the most of regulatory parameters are well identified and their estimates can be used to make conclusions about the type of gene interaction. Secondly, as parameter non-identifiability can be a consequence of their strong correlation we applied the collinearity analysis of the sensitivity matrix to reveal the subsets of correlated parameters. We found that non-identifiability of some parameters detected by the method based on confidence intervals can be explained by the correlations between different parameters. Our analysis also demonstrated that parameters of the model fitted to two genotypes are better identifiable than those of the model fitted to wild type data only.

### Mechanism of transformation of gap gene expression domains in *Kr* null mutants

Our quantitative analysis of gap gene expression in 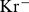 mutants confirms and extends results from earlier studies. It was previously reported that in cycle 14A the shift of posterior Gt domain is much larger than in wild type and that this domain overlaps Kni domain [15], [18]. It was also demonstrated that the level of Kni expression in *Kr* mutants is reduced. Here we showed that by gastrulation the difference in position of *gt* domain in mutants and wild type embryos constitutes approximately 10% EL. Contrary to posterior Gt domain, the anterior shift of the Kni domain maxima in *Kr* mutants constitutes only 1.8% EL, as a result these domains overlay each other. The level of kni expression remains constantly low throughout cycle 14A. Decrease in the expression levels of both *kni* and *gt* in *Kr* mutants was shown in earlier qualitative studies [15], [42], [50], [51]. High temporal resolution of our dataset enabled us to find out that the decline in gene expression level in the second half of cycle 14A turned out to be an intrinsic property of all gap domains.

**Figure 7:**
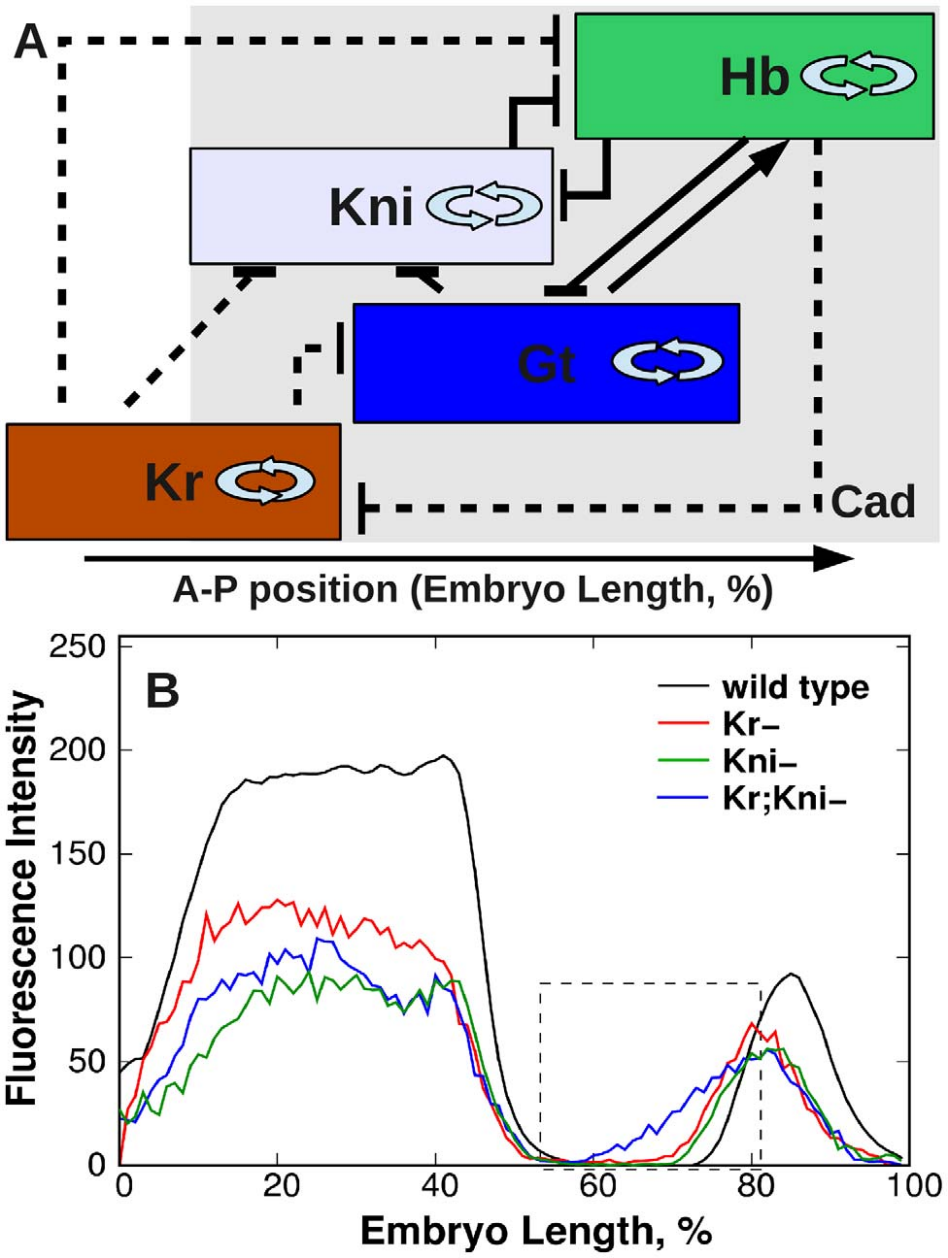
Summary of predicted gap gene regulatory mechanisms (A) and *hb* expression pattern in *Kr*, *kni*, *Kr;kni* mutants and wild type embryos from time class 6 (B). In A gap domains are shown schematically, with anterior to the left, posterior to the right. Background color indicates the most prominent activating input by Cad. Autoactivation is indicated by double arrows. T-bars indicate repressive gap-gap interactions. Dotted lines show interactions present in wild type only.

The regulatory mechanisms for expression of the trunk gap genes in the posterior of the embryo predicted by our model r summarized in Figure 7A: (1) Cad activates zygotic gap gene expression. (2) Autoactivation is involved in maintenance and sharpening of *hb*, *Kr*, *kni*, and *gt* domains. (3) Trunk gap genes either repress each other or do not interact. An important exception is activation of *hb* by Gt. (4) Bcd activates *Kr*, *gt*, *kni* in all parameter sets, however identifiability analysis showed that this activation is insignificant. In case of *hb* Bcd shows activation in approximately the same number of circuits as it shows repression. (5) In the posterior terminal region of the embryo Tll represses *Kr* and does not interact with *hb*.

In general this regulatory principles are in agreement with the results from previous studies (see Figure S4), however some differences exist. This is not surprising, if to consider that contrary to previous studies the model was fitted to two genotypes simultaneously. The rationale behind such an approach is to use the parameters of the wild type gap gene network as specific constraints on regulatory weights in mutants in order to obtain the consistent parameter estimates for both genotypes on one hand and on the other hand to preserve the characteristic features of gene regulation in mutant.

Previous models predict the mutual repression between non-overlapping gap genes *hb* and *kni*, as well as *gt* and *Kr*. Indeed, our model showed strong constraints for mutual repression between *kni* and *hb*, however identifiability analysis classified the action of Gt on *Kr* as insignificant repression. This discrepancy can be explained by the fact that in our model the parameters corresponding to *Kr* gene are estimated using data points from wild type embryos only and therefore their identifiability is inferior to that of parameters estimated from the whole dataset. The non-identifiability of parameters describing the action of Bcd on gap genes in *Kr*-deficient gap network may be explained by the exclusion from the model of the anterior half of the blastoderm, where Bcd contributes stong activating inputs on the anterior and central gap gene domains [12], [52]. Bcd activating inputs on posterior domains are smaller and the interactions of gap genes come into play to dynamically position the posterior gap domains.

In the gap gene circuit models the motif which includes Tll is the most variable component of the gap network (see Figure S4). The network reconstructed in this work constitutes no exception to this pattern: our model predicts no interaction between Tll and *hb*, while some previous models had classified this interaction as activation [31], [34] or predicted it as repression or no interaction [13], [48], [49]. In previous models Tll exerts repressive action on *gt* and *kni*, however in our model these parameters are non-identifiable. In addition our model predicts repression of *Kr* by Tll, however in the other model [34] this parameter was classified as non-identifiable.

Interestingly that in spite of the differences in gene regulation between the models discussed above the asymmetric cascade of cross-repressive interactions between gap genes with overlapping expression domains is preserved in the current two genotype model. As is evident from inspection of Table S1 the regulatory weights 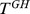 and 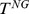 are larger than the reciprocal weights 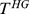 and 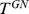 in all circuits, while the regulatory weight 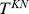 is larger than the reciprocal weight in 9 out of 11 circuits. These asymmetrical interactions lead to anterior shifts in domain positions both in wild type and mutant, as will be discussed below.

The regulatory mechanisms predicted by the model are mainly in agreement with experimental evidences. During cleavage cycle 14A both Cad and Bcd continue to activate gap genes, however the Bcd gradient starts to rapidly decay about 10–15 min before gastrulation [41], [53]. The evidence for autoactivation of gap genes is not so clear. In *Kr* and *gt* mutants expressing non-functional proteins Kr domain is narrowed and weakened [16], and the intensification of Gt domains during cycle 13 is delayed [42]. Besides computational studies predict that both Kr and Gt bind to some of their own regulatory elements [37]. The autoactivation is not required for expression of the posterior Hb domain [21]. Repressive feedback between *hb* and *kni* and *gt* and *Kr* is suggested by many experimental results [15], [42], [54]. There is experimental evidence for additional repressive interactions between gap genes with overlapping expression domains. Repression of *gt* by Hb is supported by the fact that the posterior Gt domain fails to retract from the posterior pole of the embryo around mid-cycle 14A [15], [42], while no *gt* expression can be detected in embryos over-expressing *hb* [42]. The central Kr domain expands posteriorly into regions with reduced or lacking *kni* activity in mutants [22], [55], [56]. There is a Kni binding site in the *Kr* regulatory region, which overlaps with a Bcd activator site [50]. It has been proposed that *Kr* is required for *kni* activation, however, this effect turned out to be indirect [51], [57]. *Kr* and *hb* are the only pair of overlapping gap genes that show mutual repression, however there is some ambiguity in the genetic evidence. Some authors have reported a posterior expansion of the anterior Hb domain in *Kr* mutants [54], [58]. Repression of *Kr* by Hb is suggested by an anterior expansion of the central Kr domain in *hb* mutants [22], [55], [56], [59]–[61] and multiple Hb binding sites have been identified in the *Kr* regulatory region [60]. Evidences on repression of *kni* by Gt and repression of *gt* by Kni are ambiguous [15], [42], [51], [62], e.g. a posterior expansion of the abdominal Kni domain was reported in one study [42], this effect was not seen in another [62]. Terminal gap genes have strong repressive effects on trunk gap gene *gt*, *kni* and *Kr* [38], [57], [63]. In contrast the posterior domain of Hb is present and expanded to the anterior in embryos over-expressing *tll* [64]. This suggests that Tll activates *hb* expression in its posterior domain, however, this interaction is probably indirect, since posterior Hb is present in *tll;kni* double mutants.

The important corollary that follows from the inferred topology (Figure 7A) is the prediction that in *Kr,kni* double mutants the anterior border of Hb posterior domain will not be properly set as both regulators responsible for formation of this border are deficient. We confirmed this prediction in experiment (Figure 7B). This experimental result strongly supports the model.

To explain mechanisms responsible for alteration of the gap gene expression pattern in *Kr* mutants we implement the analysis of regulatory loops and study the dynamical change of Cad contribution to a target gene regulation in wild type and mutants. Cad is the main activator of gap genes in the posterior of the embryo.

This analysis revealed two mechanisms responsible for alteration of the posterior *gt* expression pattern in *Kr* mutants. First, *gt* expression level decreases because the activating effect of Cad on *gt* diminishes with time (Figure 4). Secondly, two interactions from Kr to *gt* and *hb* responsible for formation of the anterior borders of Gt and Hb posterior domains correspondingly are impaired in 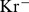 embryos (Figure 7A), that makes it possible for Gt posterior domain to move forward to the anterior due to repression by Hb.

The mechanism responsible for reduction of *kni* expression level in *Kr* mutants is essentially the same as described for *gt* posterior domain: Kni level reduces because the activating effect of Cad on *kni* decreases with time (Figure 5) and because the interaction between Kr and *gt* is deficient that makes it possible form Gt repression to spread into a territory where *kni* expression domain forms (Figure 7A).

It should be noted that the difference in Cad regulatory effect on the posterior *gt* and *kni* expression between mutant and wild type is not very large (see Figures 4C and 5C). This fact could raise doubt on the role of Cad in the reduction of gap gene expression levels in mutants. However recently it was demonstrated that only a 3% overlap exists between transcription factor occupancy and gene response to TF knockout [65], [66]. This and other results (discussed in detail in [67]) point that the relations between TF concentration and function are non-linear and that weak regulatory events and small differences in regulation may play a biologically significant role in the quantitative control of complex biological processes.

Our model can also explain the mechanism, which provides for decrease of the *hb* posterior domain expression level: such a reduction happens because Gt stops to activate *hb* due to its shift (Figure S5). However it should be noted that the activation of *hb* by Gt predicted by all gene circuit models [13], [31], [34], [48] is currently not supported by the literature.

In our model *hb* activation by Gt leads to the elevated and spurious expression of *hb* in the region of 60–77% EL (see Figure 1). The exclusion of this elevated expression from the model does not cause an increase in both *gt* and *kni* expression levels (Figure S6), that makes it unlikely that Hb repression contributes significantly to the low levels of these domains in mutant.

We note finally that the reduction of gap gene expression levels is peculiar not only for *Kr* mutants. For example *hb* expression (see Figure 7B) and *gt* posterior domain levels [26] are reduced in *kni* mutants. In wild type embryos the gap gene expression levels stop to grow in the second half of cycle 14A and slightly decrease by gastrulation [10]. Mutations in gap genes aggravate this effect, that underlines the importance of intact network for maintenance of normal gap gene expression levels.

## Methods

### Acquisition of quantitative data on gene expression

We obtained 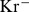 embryos from 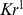 loss-of-function allele (FlyBase ID FBal0005790) [17]. 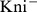 embryos were collected either from Df(3L)ri-79c or Df(3L)ri-XT1, ru[1] st[1] e[1] ca[1] stocks. *Kr;kni* double mutant embryos were made by crossing 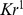 and Df(3L)ri-79c flies.

3–4 hr old embryos from flies carrying 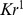 mutation [17] were collected, fixed and stained as described elsewhere [68], [69]. We used primary antibodies against Kr, Knirps (Kni), Giant (Gt), Hunchback (Hb) and Even-skipped (Eve) [68], [70] and secondary antibodies conjugated to Alexa Fluor 488, 555, 647, and 700 (Invitrogen). Each embryo was additionally stained with either anti-histone H1-4 antibody (Chemicon) or Hoechst 34580 (Invitrogen) to mark the nuclei.

Laterally oriented *Kr* null embryos, showing zero level of *Kr* expression and severely transformed Eve pattern [15], were scanned using Leica TCS SP2 and Leica TCS SP5 confocal microscopes as described [69]. For each experiment, the microscope gain and offset were set on maximum expression level of a given gene in wild type patterns and then these settings were applied for mutants. The 8-bit 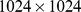 digital images of gene expression in *Kr* mutants were acquired for cleavage cycle 14A.

For spatial registration and data integration, embryos from cleavage cycle 14A were distributed into 8 time classes about 6.5 min each on the basis of measurement of degree of membrane invagination, as well as characteristic features of the *even-skipped* gene expression pattern [53]. The quantitative gene expression data and integrated patterns for each temporal class of cycle 14A were obtained as previously described [33], [69], [71], [72] using recently developed packages ProStack and BREReA [73], [74]. The one-dimensional integrated patterns of gene expression in wild type were taken from FlyEx database (http://urchin.spbcas.ru/flyex/, [75]).

### Gene circuit models

Gene circuit models [12], [13], [31], [76], [77] describe the dynamics of segmentation gene expression in the syncytial blastoderm of *Drosophila melanogaster*. The circuits used in this paper consider the time evolution of protein concentrations of gap genes *hb*, *Kr*, *gt*, and *kni* in two genotypes: wild type and in embryos with homozygous null mutation in *Kr* gene. To make separate treatment of domains with different regulatory inputs possible we narrowed down the spatial domain of the model by considering only the posterior half of the blastoderm (region from 47 to 92% embryo length (EL)), in which each of the these genes is expressed in one domain. We consider a one-dimensional row of nuclei along the anteroposterior axis of the embryo, as anteroposterior (A-P) and dorsoventral (D-V) patterning systems are largely independent of each other in the presumptive germ band of the blastoderm embryo. The modeled region extends over 45% of the A-P axis, from the minimum of *gt* expression inbetween third and fourth *gt* stripes to the posterior border of the posterior *hb* domain (Figure 1).

Gene circuits function according to three rules: interphase, mitosis and division [29]. During mitosis, only protein transport and protein decay govern the dynamics as transcription shuts down and nascent transcripts are destroyed [78]. Mitotic division is modeled as a discrete change in the state of the system. At the end of a mitosis, each nucleus is replaced with its daughter nuclei, the inter-nuclear distance is halved and the daughter nuclei inherit the state of the mother nucleus. During interphase the change in concentration 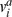 for each gap gene product *a* in each nucleus *i* over time *t* is described by the following system of ordinary differential equations (ODEs)
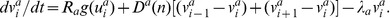
(1)


The three terms on the right-hand side of the equation represent protein synthesis, protein diffusion and protein decay. 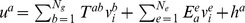 is the total regulatory input to gene *a*. 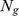 is the number of gap genes in the model (*hb*, *Kr*, *kni* and *gt*), 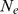 is the number of external regulatory inputs (*bcd*, *cad* and *tll* genes, which are not regulated by gap genes, but regulate these genes). 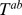 and 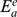 are genetic inter-connectivity matrices that characterize the action of regulator *b* or external input *e* on gene *a*. The sizes of these matrices are 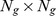 and 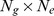 correspondingly. 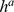 is a threshold parameter of the sigmoid regulation-expression function 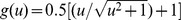. 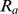 is the maximum synthesis rate, 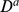 the diffusion coefficient, and 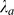 the decay rate of the product of gene *a*.

The gap gene circuits used in this study consider events occurring during cleavage cycles 13 and 14A and ending at the onset of gastrulation [3]. The divisions are carried out according to a division schedule based on experimental data. Time *t* is measured in minutes from the start of cleavage cycle 13. The interphase of cycle 13 lasts for 16.0 min, and its mitosis from 16.0 to 21.1 min. At 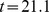 min, the thirteenth division is carried out by applying the division rule. The interphase of cycle 14A starts immediately after division, and lasts until gastrulation at 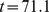 min. The cleavage cycle 14A is subdivided into 8 temporary equivalent classes, as a result the model is compared to data at 9 time points, one time point for cycle 13 (C13), and eight points for cycle 14A (time classes T1–T8).

*Kr*, *gt*, and *kni* are exclusively zygotic, and are not present at significant levels before cycle 13 [10], thus they have initial conditions of zero. For *hb*, the expression data from cycle 12 is used as the initial condition. *hb*, which is expressed both maternally and zygotically, shows a large increase in expression in cycle 13 [38], [39], indicating commencement of its zygotic expression. Non-zero initial conditions for external inputs Bcd, Cad and Tll are obtained by piecewise linear interpolation of integrated expression data at midpoint of C12 (t = −6.2 min) and midpoint of C13 (t = 10.55 min). Moreover, in order to solve the right hand side of equation (1), the concentrations of external inputs must be supplied for any time in the duration of the model. This is implemented by linear interpolation between data points at C13 and eight time classes of cycle 14A (T1–T8), with data points corresponding to midpoints of C13 and each time class.

As was suggested in previous studies [79] the zero flux boundary conditions were chosen at both ends of modeling interval because other numerically feasible alternatives, such as periodic boundary conditions, are not biological. The actual flux through the boundaries is nonzero but depends on gene expression in a complicated manner that may add the unneeded overhead for numerical simulations.

### Parameter estimation

The model parameters are estimated by fitting the model output to experimental data. This is performed by minimization of cost function based on the sum of squared differences between gap protein levels in the model and data.

DEEP - Differential Evolution Entirely Parallel method is applied to biological data fitting problem. We introduce a new migration scheme, in which the best member of a branch substitutes the oldest member of the next branch, that provides a high speed of the algorithm convergence [46].

#### Differential Evolution Entirely Parallel method

Differential Evolution (DE) is a stochastic iterative optimization technique introduced by Storn and Price [80]. It is an effective method for minimization of various and complex quality functionals. The power of DE is based on the fact that under appropriate conditions it can attain the global extremum of a functional; the weakness of this method is in high computational demand and dependence on control variables, that provides a motivation for its parallelization. Previous work in this area has produced a number of methods that perform well on particular problems.

DE starts from a set of randomly generated parameter vectors 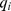, 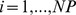. The set is called population, and vectors are called individuals. The population on each iteration is referred to as generation. The size of population *NP* is fixed. The name of the method comes from the fact that the difference between members of the current population is used to generate offsprings (see Figure S1).

Being an evolutionary algorithm, DE can be easily parallelized due to the fact that each member of population is evaluated individually. The whole population is divided into subpopulations that are sometimes called islands or branches, one per each computational node. This eliminates the restriction on the number of individuals. The individual members of branches are then allowed to migrate, i.e. move, from one branch to another according to predefined topology [81]. The number of iterations between migrations is called a communication period.

The **D**ifferential **E**volution **E**ntirely **P**arallel (DEEP) method, developed by us [46], takes into account the age of an individual that is defined as the number of iterations during which this individual survived without changes. The fact that a certain parameter set has not been updated during several iterations indicates that this set corresponds to the local minimum of the quality functional. As we seek the global minimum such a parameter set can be deleted from the population. The set of parameters corresponding to the minimal functional value found so far in a source parallel branch is copied in place of the deleted parameter set in a target branch.

The computational nodes are organized in a ring and individuals migrate from node *k* to node 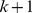 if it exists and from the last one to the first one. In [46] we have shown that the migration scheme provides a high speed of the algorithm convergence and the parallel efficiency is about 80% for the 50 nodes and 55% for 100 nodes. The reliability of the method was demonstrated by its ability to recover model parameters with about 1% accuracy.

Other details can be found in Supplementary information (See Text S1).

### Identifiability analysis

For the comprehensive analysis of modeling results it is necessary to know how reliable the parameter estimates are. In practice insufficient or noisy data, as well as the strong parameter correlation or even their functional relation may prevent the unambiguous determination of parameter values. Such parameters are related to as non-identifiable.

To reveal non-identifiable parameters the method based on confidence intervals [49], [82] is applied. The confidence intervals are constructed for the parameter estimates 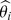 in the vicinity of model solution and are given by
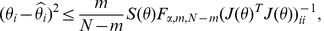
(2) where 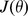 is the sensitivity matrix, the 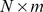 matrix of partial derivatives of the model solution with respect to the parameter vector; 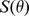 is the objective functional; 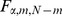 is an 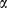-quantile of 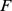-distribution with *m* and *N-m* degrees of freedom. The size of confidence intervals characterize the sensitivity of the solution to parameter changes: the shorter is the confidence interval the more reliable is the parameter estimate. If the most important feature of the parameter estimate is its sign, the identifiable estimate must have the confidence interval bounded away from zero. It is important to mention that the confidence intervals are only estimated precisely in case of independent parameters, if some parameters are strongly correlated the confidence intervals are overestimated. In other words the confidence interval (2) is the whole area of the parameter variation as the other parameters take any possible values from the *m*-dimensional confidence area (see equation (1) and Figure 1 in Text S2). Besides, correlation of parameters causes calculational errors due to ill-conditionality of the sensitivity matrix. This issue is discussed in more detail in Text S2.

The other method to detect interrelations between parameters is the collinearity analysis presented in [83]. The method is suitable for models with large number of parameters. The aim of the method is to reveal the so-called near collinear columns of the sensitivity matrix, the matrix of partial derivatives of the model solution with respect to the parameter vector, and thus detect subsets of non-identifiable parameters. Identifiability of a parameter subset is characterized by *collinearity index* defined as
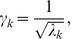
(3) where 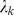 is the minimal eigenvalue of the submatrix of the Fisher information matrix. High values of 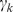 indicate that the subset of parameters is poorly identifiable due to relations between at least two parameters. The aim of the analysis is to detect all the parameter subsets of any dimension with high collinearity index such that they do not contain subsets of lower dimension for which the collinearity index is also high. Thus we reveal all the non-identifiable parameters.

For more detailed description of the methods see Text S2.

## Supporting Information

Figure S1 Geometric interpretation of Differential Evolution.
(PDF)

Figure S295% confidence intervals for estimates of regulatory weights, elements of genetic inter-connectivity matrix *T* in 11 circuits. Regulators and target genes are gap genes *hb* (H), *Kr*(K), *gt*(G) and *kni*(N). Graphs are labeled by gene notations, the first letter corresponds to the target gene (e.g., HK stands for 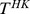).(PDF)

Figure S395% confidence intervals for estimates of elements of genetic inter-connectivity matrix *E* in 11 circuits. Target genes are gap genes *hb* (H), *Kr*(K), *gt*(G) and *kni*(N); external regulators are *bcd*(B), *cad*(C) and *tll*(T). Graphs are labeled by gene notations, the first letter corresponds to the target gene (e.g., HB stands for 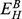).(PDF)

Figure S4 Comparison of the gap gene network topologies predicted by the current two genotype model (A) and earlier models (B). Dashed lines show interactions with regulatory weights that were either non-identifiable or classified into different categories in different models.
(PDF)

Figure S5Interactions involved in regulation of Hb posterior domain. A. Modeled expression patterns at temporal classes T1, T3 and T7. B. Regulatory contributions in wild type (center) and mutant (right). C. Temporal change in regulatory contributions at position corresponding to Hb domain maximum. Colored areas are given by 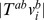 or 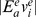 in equation (1) and reflect the strength of a given interaction at a specific point in space and time. All plots are based on best scoring solution (circuit C9, see Table S1 for parameters).(PDF)

Figure S6 Spurious expression of *hb* in the region of 60–77% EL is not responsible for decrease in the *gt* expression level. Patterns for mutant without the spurious domain shown in the right panel were obtained with Gt input to *hb* expression equal to zero. To compensate the decrease in repression level we set the *gt* and *kni* autoactivation to 70% of values found by fitting the model to data. The left panel is shown for comparison; it demonstrates that in wild type embryos the autoactivation level decrease does not lead to reduction of *gt* and *kni* expression to the levels observed in mutants.
(PDF)

Table S1Parameters of a representative gene network. Rows correspond to target genes, columns to regulators *hb* (H), *Kr*(K), *gt*(G), *kni*(N), *bcd*(B), *cad*(C) and *tll*(T). *R* - maximum synthesis rate, *D* - diffusion coefficient, 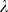 - decay rate, 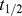 is a protein half-life measured in minutes. Promoter thresholds *h* for all genes were fixed at value −3.5. This reduces time needed for DEEP to find the minimum. The values for diffusion coefficient *D* are given as they appear in the equations so the units are 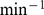. To relate these values to conventional diffusion coefficient, we should consider the diffusion term in the model equations as a finite difference approximation of the conventional diffusion term containing the second order spatial derivative. The approximation takes place at the 1D mesh of points that coincide with real nuclei. That gives the following approximate formula for the conventional diffusion coefficient 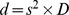, where 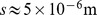 is the distance between adjacent nuclei. As the real diffusion coefficients of the proteins in question haven't been measured in live embryos or such measurements are not available we can compare our values with results for other proteins. For example, the diffusion coefficient of Bcd was measured in live *Drosophila* embryos by Gregor et al. [85] as 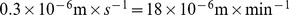. By using the above formula, we get the following values for the conventional diffusion coefficient for gap proteins obtained from the fitting: 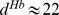, 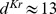, 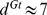, and 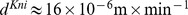. These values are realistic.(PDF)

Table S2 Circuit parameter sets: 11 sets of parameter values that optimize the transformed functional. Regulatory weights are labeled by single-letter notations of genes: *hb*(H), *Kr*(K), *gt*(G), *kni*(N), *bcd*(B), *cad*(C), *tll*(T).
(PDF)

Text S1 Supplementary methods. Parameter estimation.
(PDF)

Text S2 Supplementary methods. Identifiability analysis.
(PDF)
