# Supplementary figures and images for: Modeling of Gap Gene Expression in Drosophila Kruppel Mutants
Source: PLoS Comput Biol. 2012 Aug 23;8(8):e1002635. doi: 10.1371/journal.pcbi.1002635 (PMC3426564; doi:10.1371/journal.pcbi.1002635)

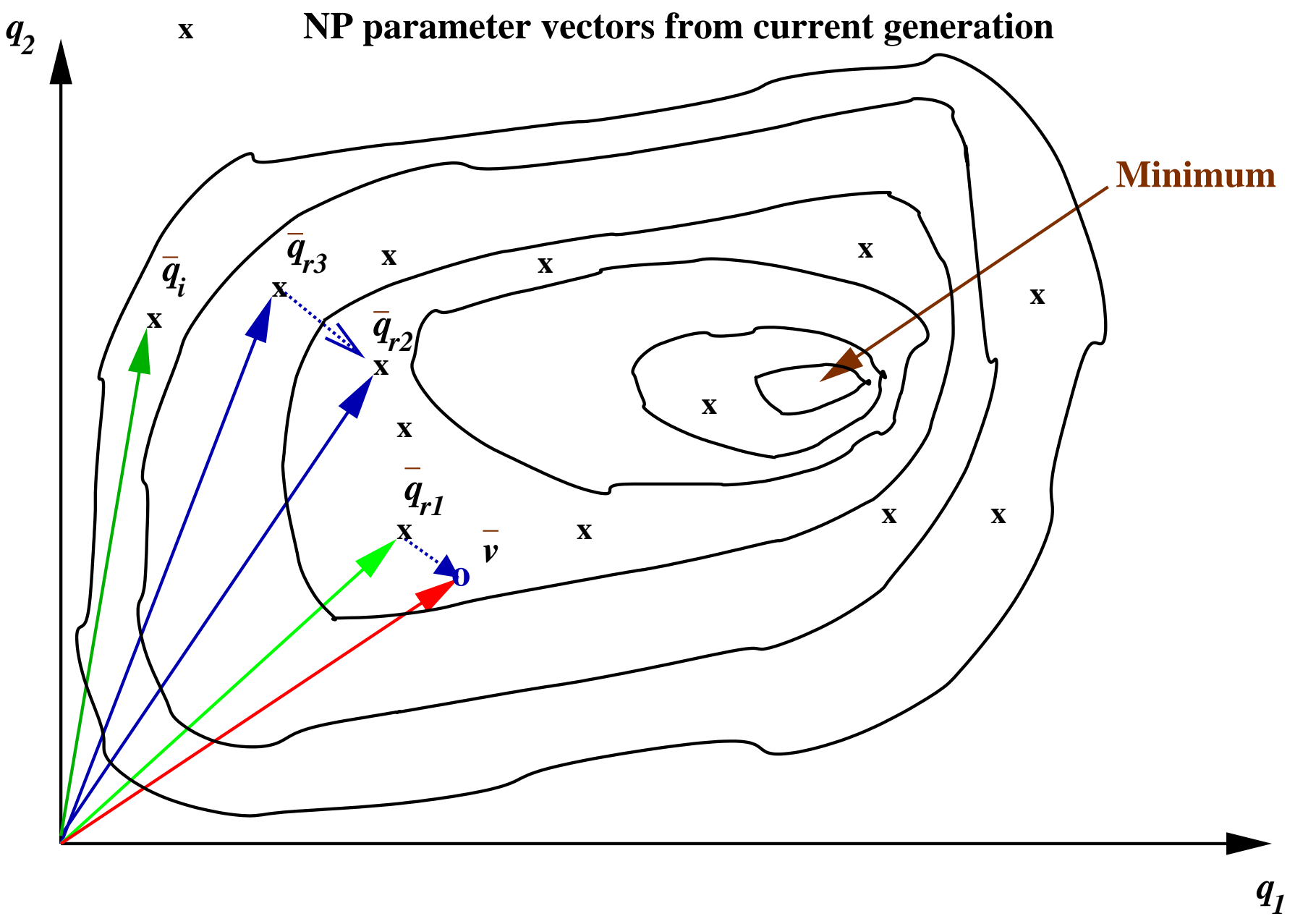

Supplement: Figure S1 — Geometric interpretation of Differential Evolution. (PDF) [file pcbi.1002635.s001.pdf]

Estimate of regulatory weight

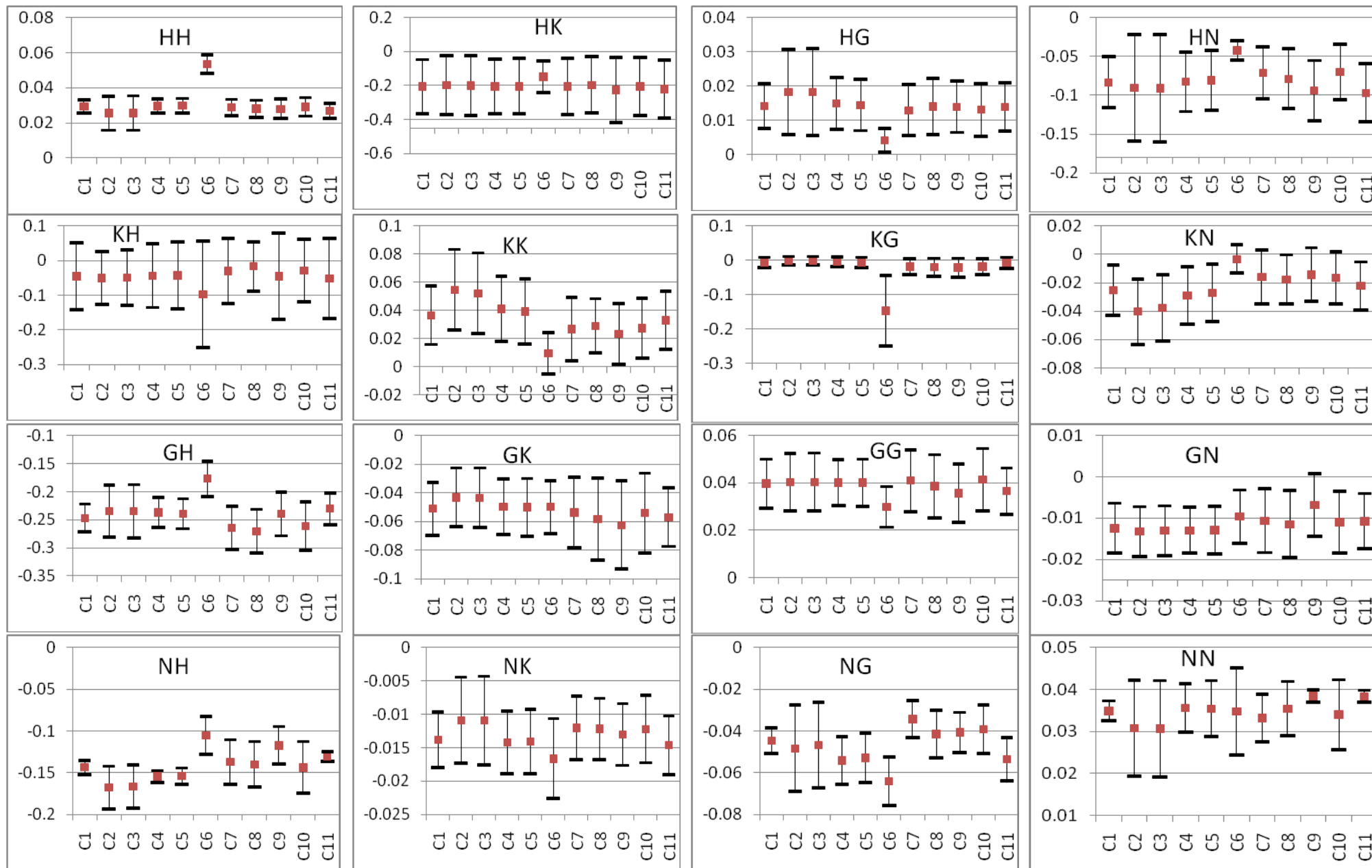

Circuits

Supplement: Figure S2 — 95% confidence intervals for estimates of regulatory weights, elements of genetic inter-connectivity matrix T in 11 circuits. Regulators and target genes are gap genes hb (H), Kr(K), gt(G) and kni(N). Graphs are labeled by gene notations, the first letter corresponds to the target gene (e.g., HK stands for ). (PDF) [file pcbi.1002635.s002.pdf]

Estimate of regulatory weight

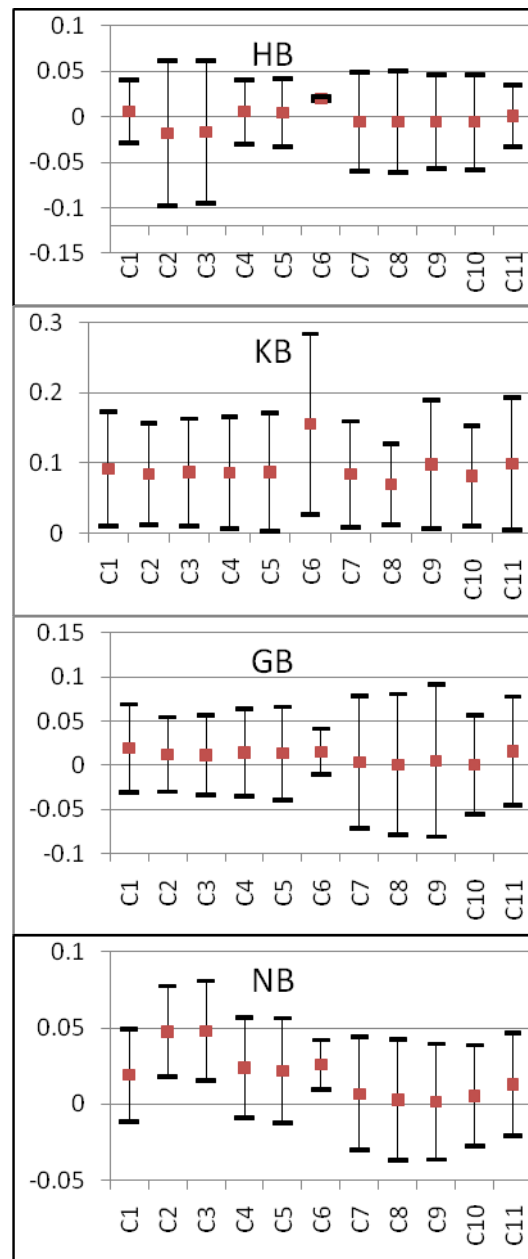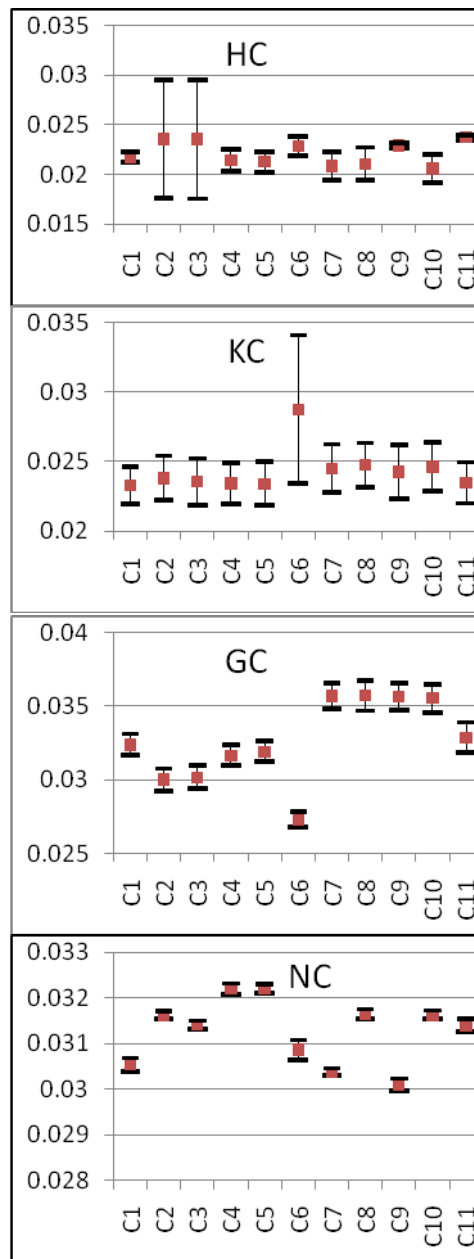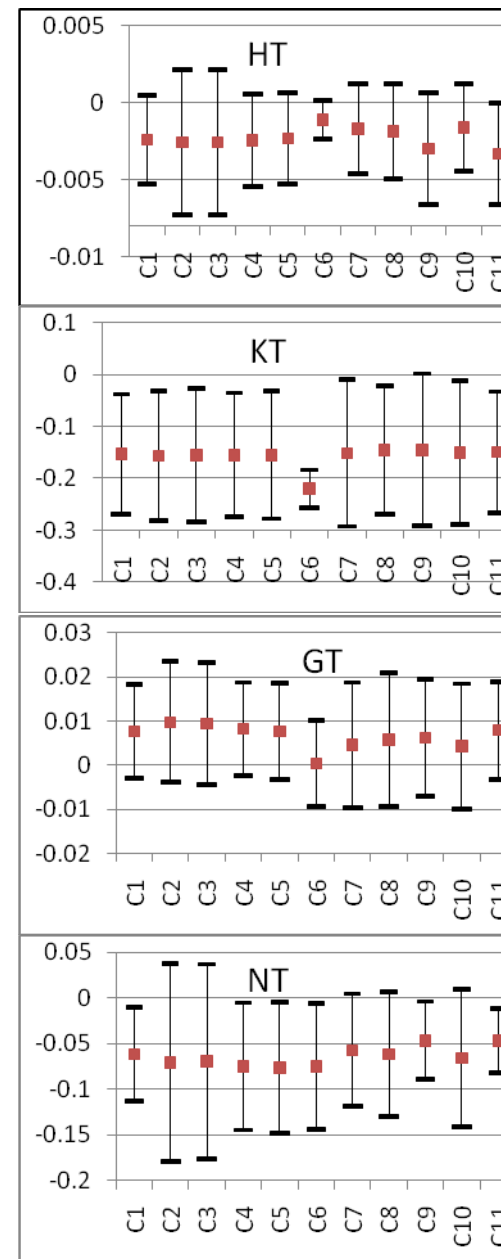

Circuits

Supplement: Figure S3 — 95% confidence intervals for estimates of elements of genetic inter-connectivity matrix E in 11 circuits. Target genes are gap genes hb (H), Kr(K), gt(G) and kni(N); external regulators are bcd(B), cad(C) and tll(T). Graphs are labeled by gene notations, the first letter corresponds to the target gene (e.g., HB stands for ). (PDF) [file pcbi.1002635.s003.pdf]

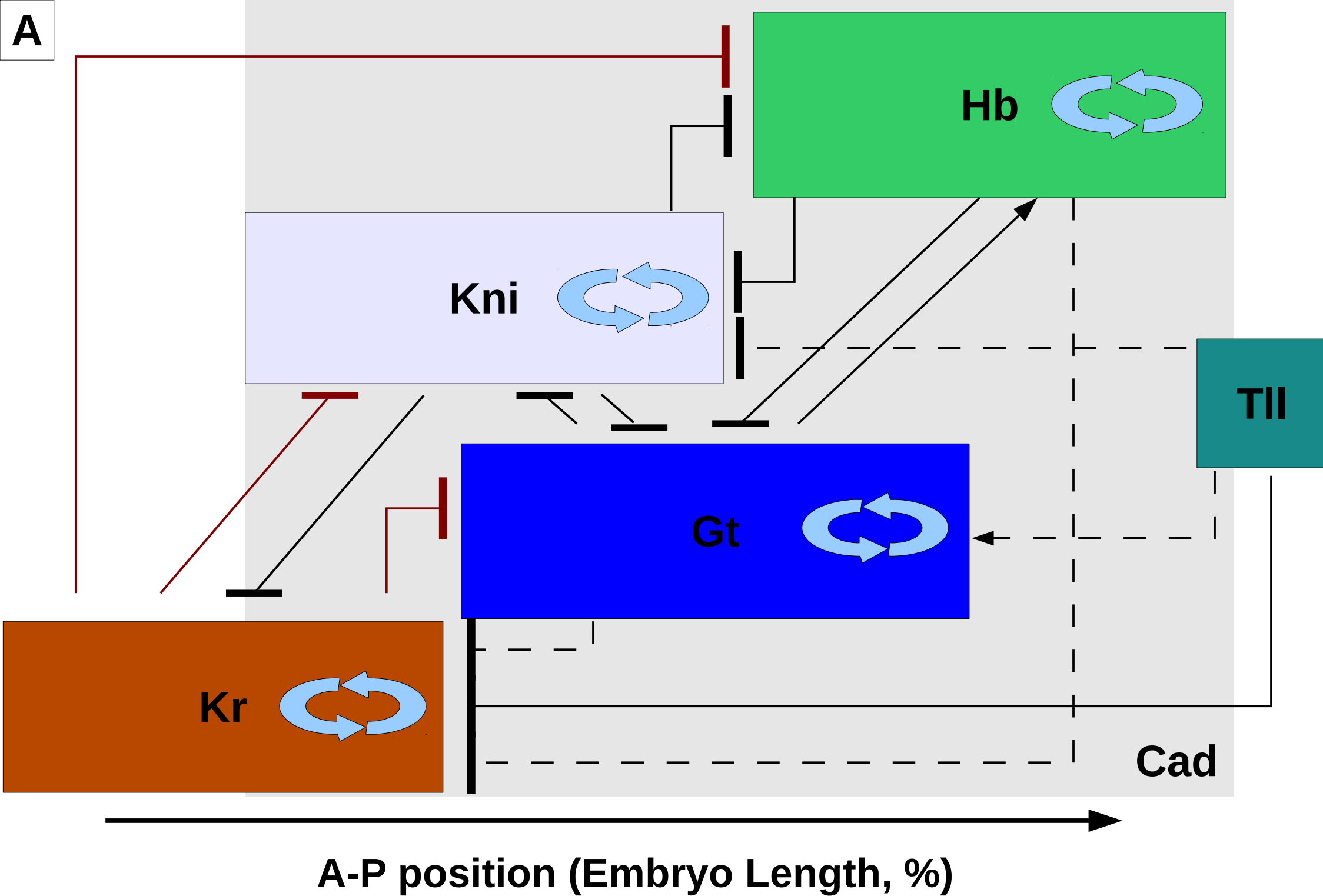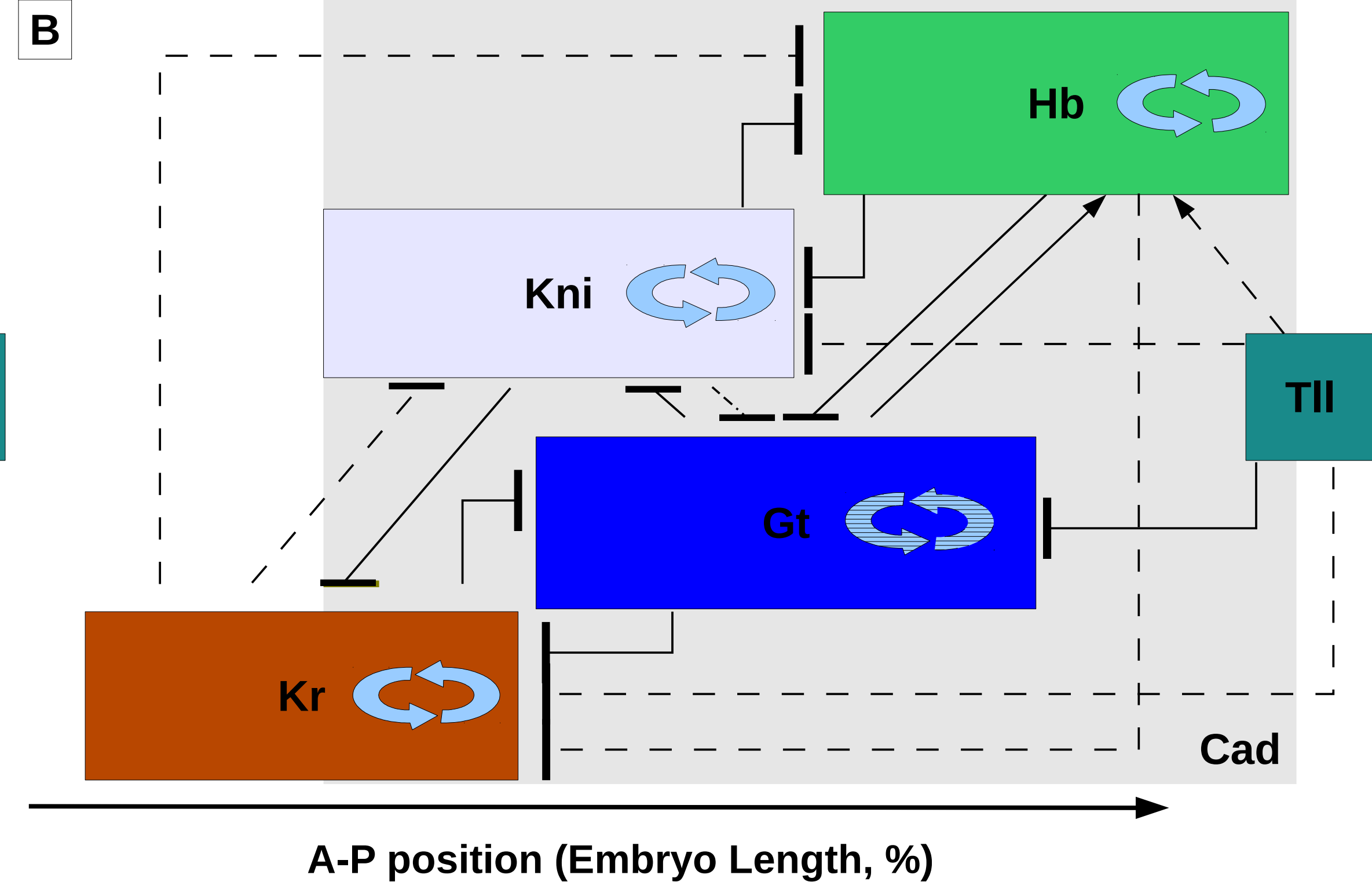

Supplement: Figure S4 — Comparison of the gap gene network topologies predicted by the current two genotype model (A) and earlier models (B). Dashed lines show interactions with regulatory weights that were either non-identifiable or classified into different categories in different models. (PDF) [file pcbi.1002635.s004.pdf]

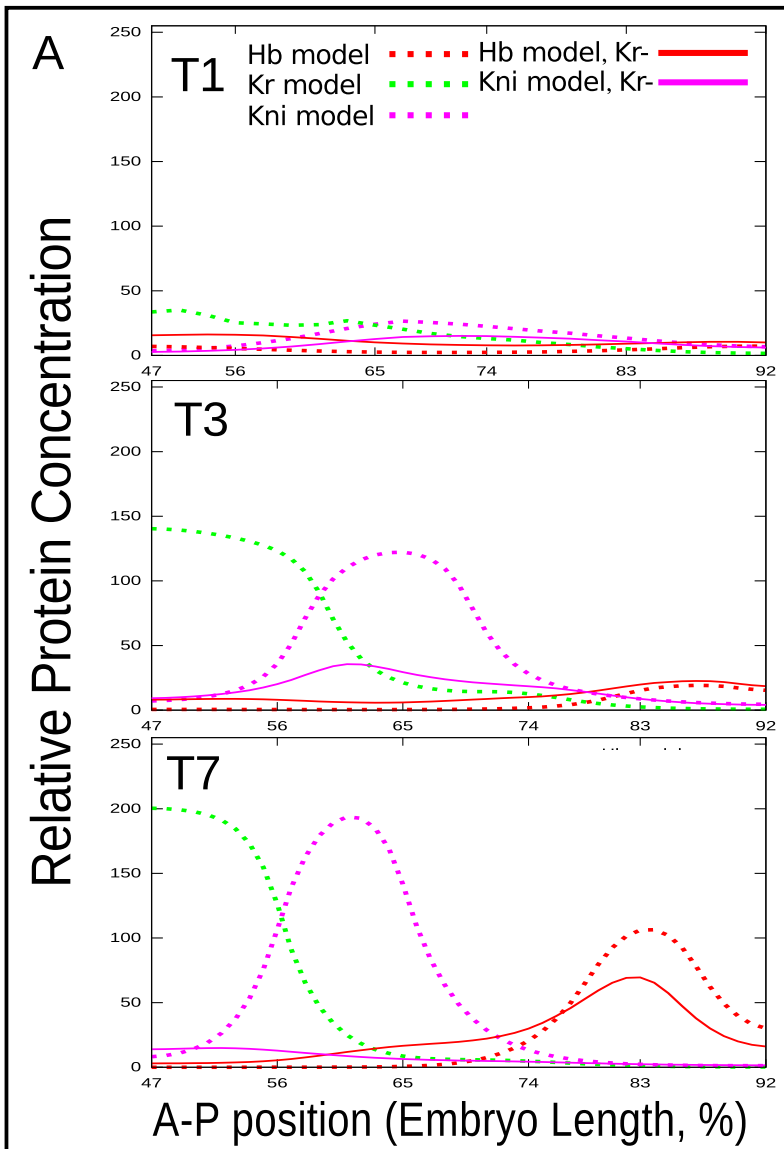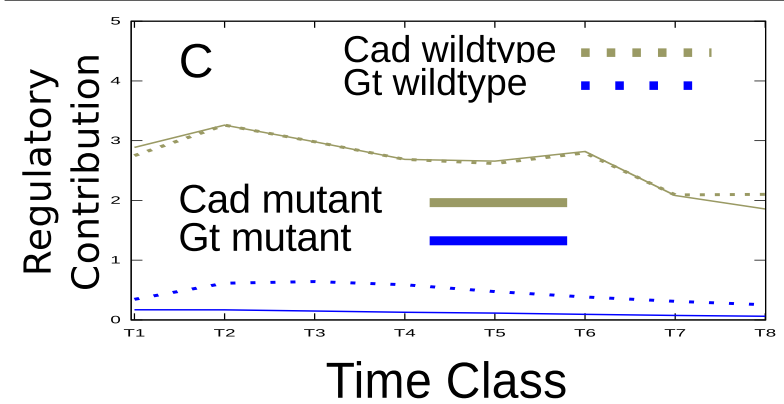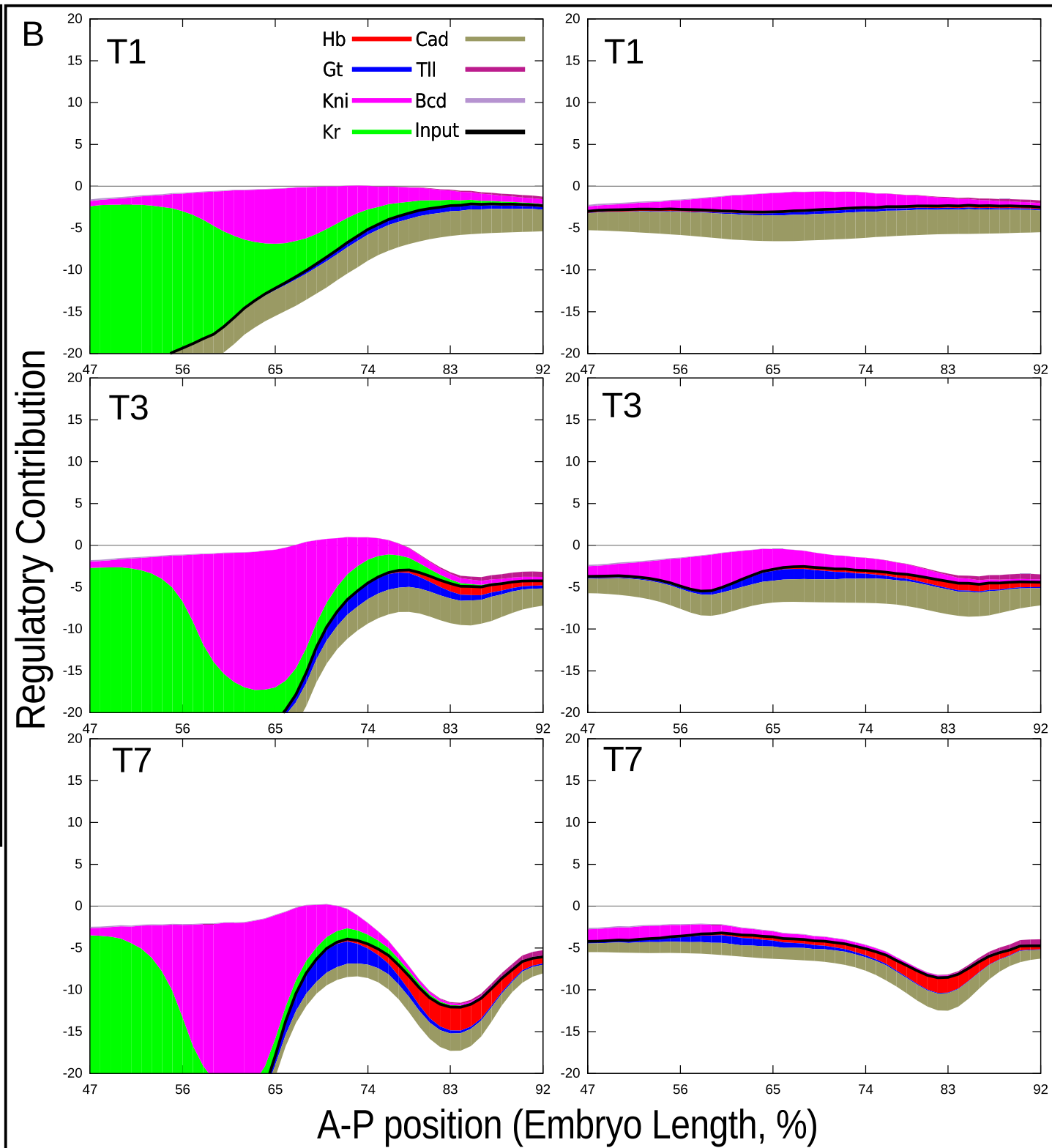

Supplement: Figure S5 — Interactions involved in regulation of Hb posterior domain. A. Modeled expression patterns at temporal classes T1, T3 and T7. B. Regulatory contributions in wild type (center) and mutant (right). C. Temporal change in regulatory contributions at position corresponding to Hb domain maximum. Colored areas are given by or in equation (1) and reflect the strength of a given interaction at a specific point in space and time. All plots are based on best scoring solution (circuit C9, see Table S1 for parameters). (PDF) [file pcbi.1002635.s005.pdf]

Relative Protein Concentration

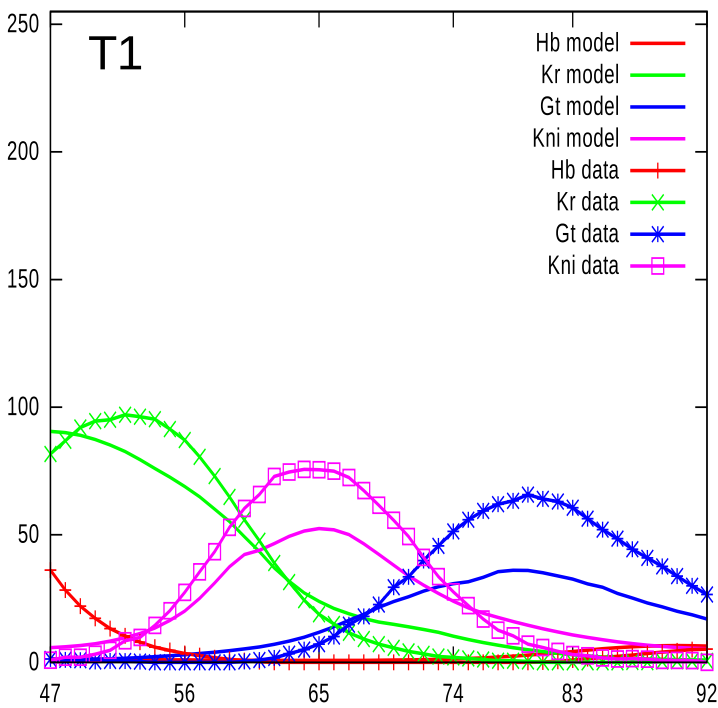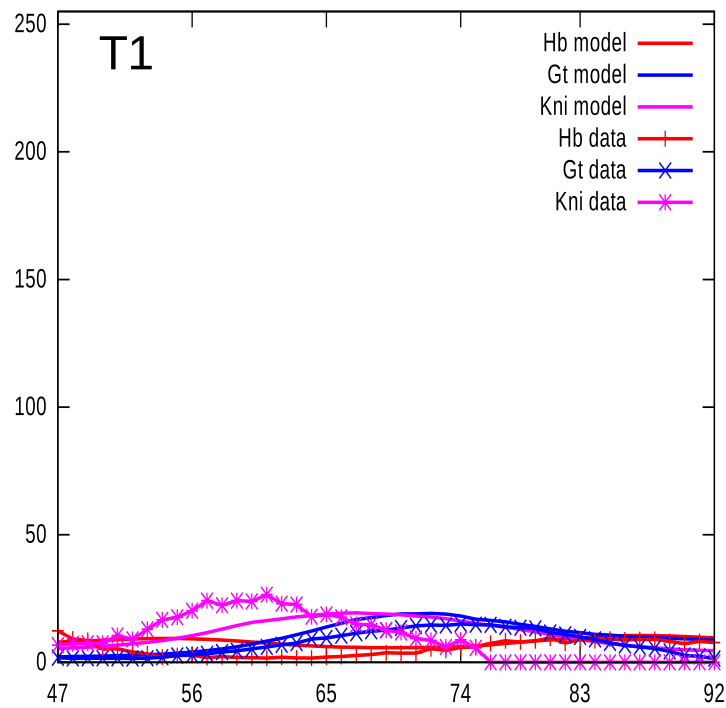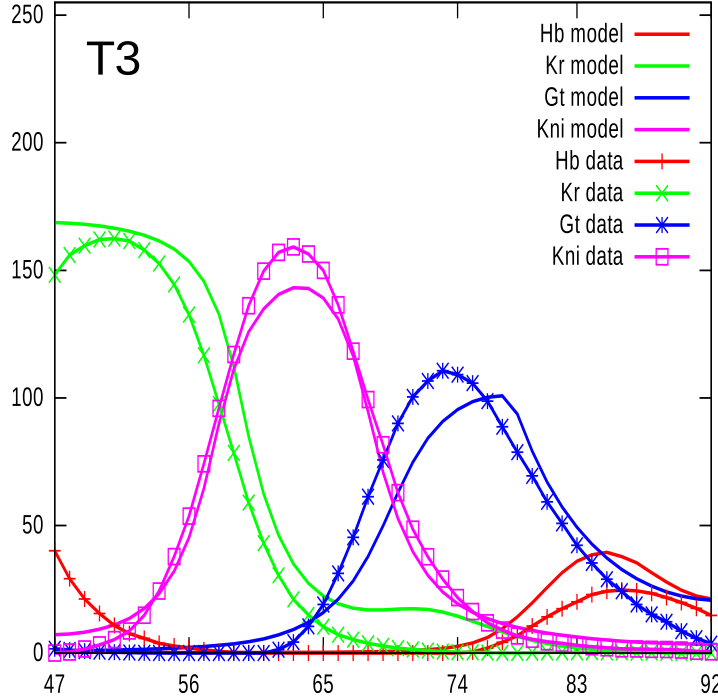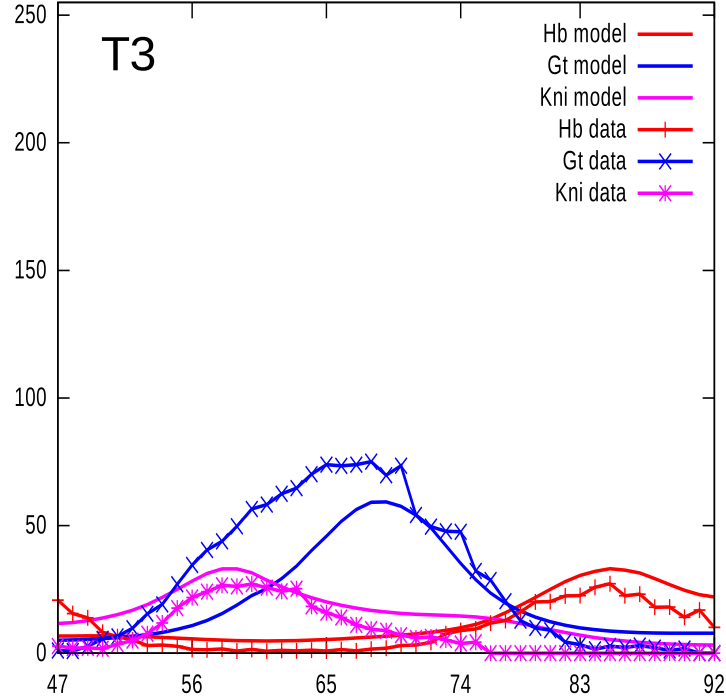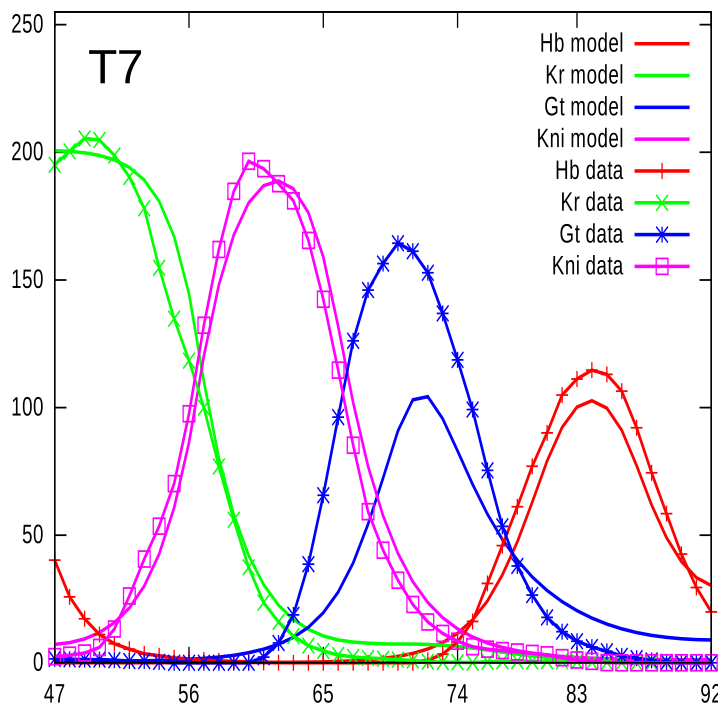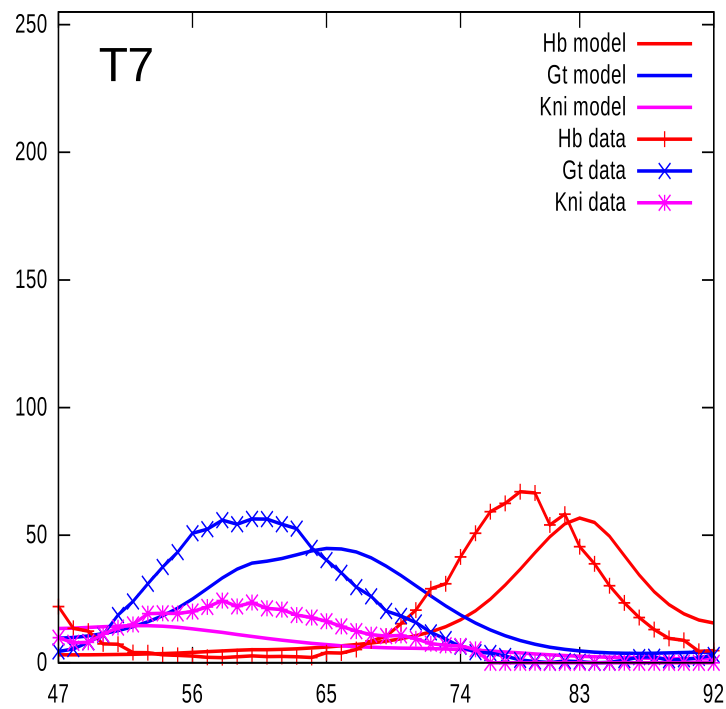

A-P position (Embryo Length, %)

Supplement: Figure S6 — Spurious expression of hb in the region of 60–77% EL is not responsible for decrease in the gt expression level. Patterns for mutant without the spurious domain shown in the right panel were obtained with Gt input to hb expression equal to zero. To compensate the decrease in repression level we set the gt and kni autoactivation to 70% of values found by fitting the model to data. The left panel is shown for comparison; it demonstrates that in wild type embryos the autoactivation level decrease does not lead to reduction of gt and kni expression to the levels observed in mutants. (PDF) [file pcbi.1002635.s006.pdf]
