# Supplementary material for: Modeling of Gap Gene Expression in Drosophila Kruppel Mutants
Source: PLoS Comput Biol. 2012 Aug 23;8(8):e1002635. doi: 10.1371/journal.pcbi.1002635 (PMC3426564; doi:10.1371/journal.pcbi.1002635)
Supplement: Table S1 — Parameters of a representative gene network. Rows correspond to target genes, columns to regulators hb (H), Kr(K), gt(G), kni(N), bcd(B), cad(C) and tll(T). R - maximum synthesis rate, D - diffusion coefficient, - decay rate, is a protein half-life measured in minutes. Promoter thresholds h for all genes were fixed at value −3.5. This reduces time needed for DEEP to find the minimum. The values for diffusion coefficient D are given as they appear in the equations so the units are . To relate these values to conventional diffusion coefficient, we should consider the diffusion term in the model equations as a finite difference approximation of the conventional diffusion term containing the second order spatial derivative. The approximation takes place at the 1D mesh of points that coincide with real nuclei. That gives the following approximate formula for the conventional diffusion coefficient , where is the distance between adjacent nuclei. As the real diffusion coefficients of the proteins in question haven't been measured in live embryos or such measurements are not available we can compare our values with results for other proteins. For example, the diffusion coefficient of Bcd was measured in live Drosophila embryos by Gregor et al. [85] as . By using the above formula, we get the following values for the conventional diffusion coefficient for gap proteins obtained from the fitting: , , , and . These values are realistic. (PDF) [file pcbi.1002635.s007.pdf]

Target	R	*hb*	*Kr*	*gt*	*kni*	*bcd*	*cad*	*tll*	h	D	$t_{1/2}=\log(2) / \lambda$
hb	20.000	0.026	-0.237	0.017	-0.104	-0.009	0.026	-0.004	-3.500	0.895	5.569
Kr	20.000	-0.055	0.029	-0.005	-0.018	0.108	0.022	-0.156	-3.500	0.519	7.176
gt	10.000	-0.251	-0.072	0.036	-0.008	0.015	0.036	0.006	-3.500	0.280	15.507
kni	18.162	-0.128	-0.016	-0.050	0.036	0.006	0.031	-0.031	-3.500	0.627	9.358
