# Supplementary material for: Modeling of Gap Gene Expression in Drosophila Kruppel Mutants
Source: PLoS Comput Biol. 2012 Aug 23;8(8):e1002635. doi: 10.1371/journal.pcbi.1002635 (PMC3426564; doi:10.1371/journal.pcbi.1002635)
Supplement: Table S2 — Circuit parameter sets: 11 sets of parameter values that optimize the transformed functional. Regulatory weights are labeled by single-letter notations of genes: hb(H), Kr(K), gt(G), kni(N), bcd(B), cad(C), tll(T). (PDF) [file pcbi.1002635.s008.pdf]

Θ^{hb}	T^{HH}	T^{HK}	T^{HG}	T^{HN}	E_H^B	E_H^C	E_H^T	R_{hb}	D^{hb}	λ^{hb}
C1	0.029	-0.206	0.014	-0.083	0.006	0.022	-0.002	19.93	0.819	0.122
C2	0.025	-0.198	0.018	-0.090	-0.019	0.024	-0.003	10.00	0.325	0.051
C3	0.026	-0.200	0.018	-0.091	-0.017	0.024	-0.003	10.00	0.324	0.051
C4	0.030	-0.205	0.015	-0.083	0.005	0.021	-0.002	19.84	0.834	0.120
C5	0.030	-0.205	0.014	-0.081	0.005	0.021	-0.002	19.87	0.848	0.121
C6	0.053	-0.149	0.004	-0.042	0.020	0.023	-0.001	10.02	0.181	0.133
C7	0.029	-0.205	0.013	-0.071	-0.005	0.021	-0.002	19.71	0.542	0.115
C8	0.028	-0.196	0.014	-0.079	-0.006	0.021	-0.002	19.82	0.543	0.114
C9	0.028	-0.226	0.014	-0.094	-0.005	0.023	-0.003	20.00	0.876	0.111
C10	0.029	-0.205	0.013	-0.070	-0.006	0.021	-0.002	19.69	0.528	0.116
C11	0.027	-0.221	0.014	-0.097	0.001	0.024	-0.003	20.00	0.911	0.116

Θ^{Kr}	T^{KH}	T^{KK}	T^{KG}	T^{KN}	E_K^B	E_K^C	E_K^T	R_{Kr}	D^{Kr}	λ^{Kr}
C1	-0.045	0.036	-0.007	-0.025	0.092	0.023	-0.153	19.93	0.318	0.103
C2	-0.050	0.055	-0.001	-0.040	0.084	0.024	-0.157	19.87	0.425	0.105
C3	-0.050	0.052	-0.001	-0.038	0.087	0.024	-0.155	19.95	0.430	0.105
C4	-0.044	0.041	-0.004	-0.029	0.086	0.023	-0.155	19.86	0.353	0.102
C5	-0.043	0.039	-0.006	-0.027	0.087	0.023	-0.155	19.93	0.344	0.103
C6	-0.098	0.009	-0.147	-0.003	0.155	0.029	-0.220	20.00	0.294	0.090
C7	-0.030	0.027	-0.018	-0.016	0.084	0.024	-0.151	20.00	0.255	0.104
C8	-0.017	0.029	-0.020	-0.018	0.070	0.025	-0.146	19.77	0.257	0.103
C9	-0.045	0.023	-0.022	-0.014	0.098	0.024	-0.146	19.93	0.187	0.102
C10	-0.028	0.027	-0.019	-0.017	0.081	0.025	-0.151	19.92	0.256	0.104
C11	-0.052	0.033	-0.008	-0.022	0.099	0.023	-0.150	19.90	0.262	0.104

Θ^{gt}	T^{GH}	T^{GK}	T^{GG}	T^{GN}	E_G^B	E_G^C	E_G^T	R_{gt}	D^{gt}	λ^{gt}
C1	-0.247	-0.051	0.040	-0.012	0.019	0.032	0.008	13.45	0.414	0.065
C2	-0.234	-0.043	0.040	-0.013	0.012	0.030	0.010	15.32	0.616	0.069
C3	-0.235	-0.044	0.040	-0.013	0.011	0.030	0.009	15.17	0.614	0.069
C4	-0.237	-0.050	0.040	-0.013	0.014	0.032	0.008	14.00	0.471	0.066
C5	-0.239	-0.050	0.040	-0.013	0.013	0.032	0.008	13.81	0.471	0.065
C6	-0.177	-0.050	0.030	-0.010	0.015	0.027	0.000	20.00	0.840	0.082
C7	-0.265	-0.054	0.041	-0.011	0.003	0.036	0.005	10.00	0.232	0.054
C8	-0.271	-0.058	0.039	-0.011	0.001	0.036	0.006	10.36	0.270	0.049
C9	-0.240	-0.063	0.036	-0.007	0.005	0.036	0.006	10.01	0.174	0.054
C10	-0.261	-0.054	0.041	-0.011	0.000	0.036	0.004	10.00	0.255	0.054
C11	-0.230	-0.057	0.036	-0.011	0.016	0.033	0.008	13.55	0.406	0.065

Θ^{kni}	T^{NH}	T^{NK}	T^{NG}	T^{NN}	E_N^B	E_N^C	E_N^T	R_{kni}	D^{kni}	λ^{kni}
C1	-0.144	-0.014	-0.045	0.035	0.019	0.031	-0.062	20.00	0.668	0.089
C2	-0.168	-0.011	-0.048	0.031	0.048	0.032	-0.071	15.81	0.477	0.067
C3	-0.166	-0.011	-0.047	0.0311	0.048	0.031	-0.070	15.96	0.481	0.067
C4	-0.155	-0.014	-0.054	0.036	0.024	0.032	-0.075	16.78	0.542	0.073
C5	-0.154	-0.014	-0.053	0.035	0.022	0.032	-0.077	16.87	0.545	0.074
C6	-0.105	-0.017	-0.064	0.035	0.026	0.031	-0.075	16.60	0.545	0.065
C7	-0.137	-0.012	-0.034	0.033	0.007	0.030	-0.057	19.92	0.589	0.091
C8	-0.140	-0.012	-0.042	0.035	0.003	0.032	-0.062	16.96	0.529	0.079
C9	-0.117	-0.013	-0.041	0.038	0.002	0.030	-0.047	18.14	0.617	0.084
C10	-0.144	-0.012	-0.039	0.034	0.005	0.032	-0.066	17.35	0.505	0.079
C11	-0.131	-0.015	-0.053	0.038	0.013	0.031	-0.047	17.74	0.600	0.080
