# Supplementary material for: Modeling of Gap Gene Expression in Drosophila Kruppel Mutants
Source: PLoS Comput Biol. 2012 Aug 23;8(8):e1002635. doi: 10.1371/journal.pcbi.1002635 (PMC3426564; doi:10.1371/journal.pcbi.1002635)
Supplement: Text S1 — Supplementary methods. Parameter estimation. (PDF) [file pcbi.1002635.s009.pdf]

1 Parameter estimation

1.1 Formal problem statement

Let the vector $v(t,q) = (v_0(t,q), ..., v_{K-1}(t,q))^T$ describe the state of the system at time t. The dimension of v equals the number of state variables K. Let the vector $q = (q_0, ..., q_{I-1})^T$ be the vector of parameters of dimension I.

The system of ordinary differential equations of the first order with respect to the independent variable t and initial conditions describe the dynamics of the system:

$$\frac{\partial v}{\partial t} = f(v,q); \qquad v(0,q) = v_0; \tag{1.1}$$

The model parameters are estimated by fitting the model output to experimental data. This is performed by minimization of the quality functional that is defined as the sum of squared differences between the data and model output:

$$\begin{aligned} F(v,q) = \sum_{i=1}^{J} (v(t_i,q) - y(t_i))^T (v(t_i,q) - y(t_i)) + P(q) \\ = \varphi(v_1, ..., v_J) + P(q) \rightarrow \min \\ v_i = v(t_i,q), \end{aligned} \tag{1.2}$$

where J independent experimental observations are denoted as $y(t_i) = (y_0(t), ..., y_{K-1}(t))^T$ and $i = 1, ..., J$.

Inequality constraints are imposed on the subset of parameters:

$$q_i^{low} \leq q_i \leq q_i^{up} \quad i \in I_l. \tag{1.3}$$

System (1.1) is sometimes referred to as differential constraint. Problems of mathematical physics described by differential equations of higher orders can be rewritten in *normal* form, like (1.1)[1].

1.2 Calculation of trial vectors

The first trial vector is calculated by

$$v = q_{r1} + S(q_{r2} - q_{r3}) \tag{1.4}$$

where $q_\bullet$ is a member of the current generation g, S is a predefined scaling constant and $r1$, $r2$, $r3$ are different random indices of the members of population. The second trial vector is calculated using "trigonometric mutation rule" [2]

$$\begin{aligned} z &= \frac{q_{r1} + q_{r2} + q_{r3}}{3} + (\varphi_2 - \varphi_1)(q_{r1} - q_{r2}) \\ &+ (\varphi_3 - \varphi_2)(q_{r2} - q_{r3}) + (\varphi_1 - \varphi_3)(q_{r3} - q_{r1}) \end{aligned} \quad (1.5)$$

where $\varphi_i = |F(q_{ri})|/\varphi^*$, $i = 1, 2, 3$, $\varphi^* = |F(q_{r1})| + |F(q_{r2})| + |F(q_{r3})|$. The third trial vector is defined as

$$w_j = \begin{cases} v_j, & j = \langle n \rangle_I, \langle n+1 \rangle_I, ..., \langle n+L-1 \rangle_I \\ z_j & j < \langle n \rangle_I \; OR \; j > \langle n+L-1 \rangle_I \end{cases} \quad (1.6)$$

where n is a randomly chosen index, $\langle x \rangle_y$ is the remainder of division x by y, and L is determined by $Pr(L = a) = (p)^a$, where p is the probability of crossover. The new individuum replaces it's parent if the value of the quality functional for it's set of parameters is less than that for the current one.

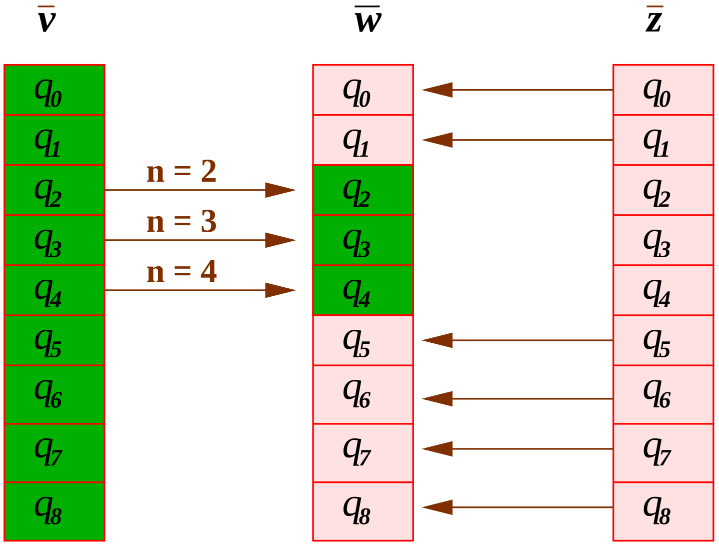

Figure 1: Construction of the trial vector

The process is illustrated in Fig. 1.

1.3 Preserving population diversity

The original algorithm was highly dependent on internal parameters as reported by other authors, see, for example [3]. An efficient adaptive scheme for selection of internal parameters S and p based on the control of the population diversity was proposed in [4]:

$$var_j = \frac{1}{NP}\sum_{i=0}^{NP-1}\left(q_{i,j} - \frac{1}{NP}\sum_{k=0}^{NP-1} q_{k,j}\right)^2 \tag{1.7}$$

where $j = 0, ..., I-1$. Then

$$S_j = \begin{cases} \sqrt{\frac{NP\cdot(c_j-1)+p_j(2-p_j)}{2\cdot NP\cdot p_j}} & NP\cdot(c_j-1)+p_j(2-p_j)\geq 0 \\ S_{inf} & NP\cdot(c_j-1)+p_j(2-p_j) < 0 \end{cases} \tag{1.8}$$

and

$$p_j = \begin{cases} -(NP\cdot S_j^2-1)+\sqrt{(NP\cdot S_j^2-1)^2 - NP\cdot(1-c_j)} & c_j \geq 1 \\ p_{inf} & c_j < 1 \end{cases} \tag{1.9}$$

and a new control parameter γ was introduced

$$c_j^{new} = \gamma\left(var_j / var_j^{new}\right) \tag{1.10}$$

1.4 Differential Evolution Entirely Parallel method

Being an evolutionary algorithm, DE can be easily parallelized due to the fact that each member of the population is evaluated individually. Two approaches have been developed:

- Each individuum is assigned to one node;
- The whole population is divided into subpopulations that are sometimes called islands or branches, one per each computational node.

The second approach eliminates the restriction on the number of individuals (see 3). Individual members of branches are then allowed to migrate, i.e. move, from one branch to another according to predefined topology [5]. The number of iterations between migrations is called communication period Π.

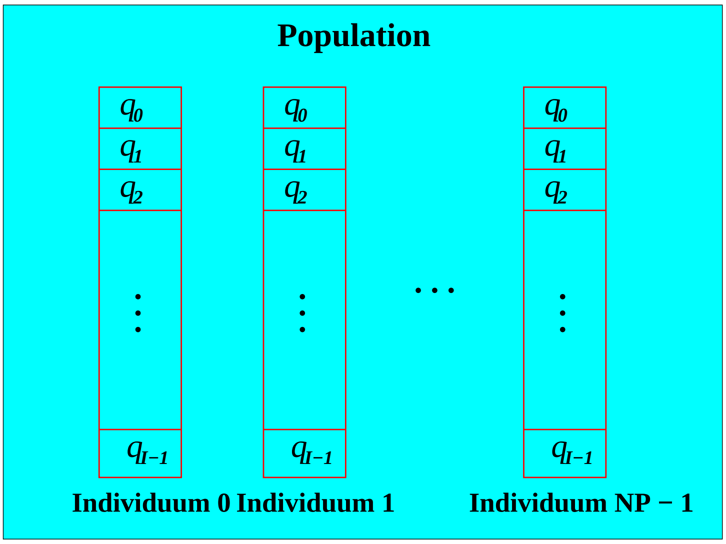

Figure 2: Differential Evolution processes the population of individuals

The **D**ifferential **E**volution **E**ntirely **P**arallel (DEEP) method, developed by authors [6] takes into account the age of individuals during the evolution. The age is defined as the number of method iterations that this individual survived without changes. The fact that the certain parameter set has not been updated during several iterations indicates that this set corresponds to the local minimum of the quality functional. As we seek the global minimum such parameter set can be deleted from the population. The set of parameters that corresponds to the minimal functional value found by the moment in parallel branch is copied to replace the deleted parameter set in target branch.

The computational nodes are organized in a ring(see Fig. 4) and individuals migrate from node k to node $k+1$, if it exists, and from the last one to the first one. The migration scheme provides a high speed of the algorithm convergence.

1.5 Trigonometric transformation of constraints

Let us introduce new parameters u_i

$$q_i = \alpha_i + \beta_i \ \sin \ (\eta u_i), \tag{1.11}$$

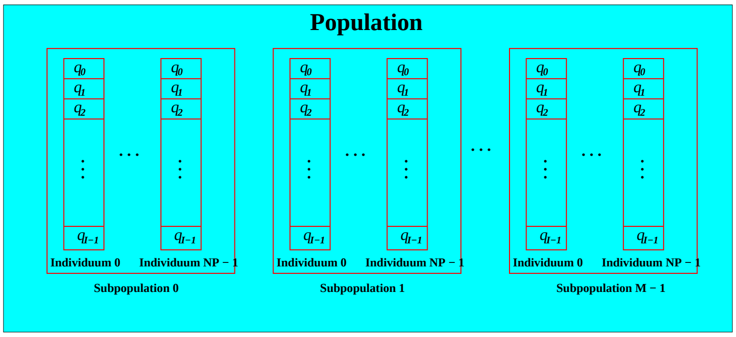

Figure 3: The whole population is subdivided into branches

where scaling coefficient η is chosen experimentally, and

$$\alpha_i = (q_i^{up} + q_i^{low})/2; \quad \beta_i = (q_i^{up} - q_i^{low})/2.$$

Another transformation can be also used

$$q_i = \alpha_i + \beta_i \ \tanh\ (\eta u_i), \tag{1.12}$$

Consequently, parameters q_i, $i \in I_l$ in (1.1) are substituted with their transformations (1.11) or (1.12), and DEEP is applied to determine unconstrained parameters u_i.

1.6 Stopping criterion

Calculations are stopped in case that the functional F decreases less than a predefined value ρ during M steps, Fig. 5.

1.7 Implementation

The serial algorithm was implemented in ANSI C programming language and run on Dell PowerEdge 2800 with 2 Xeons 2.4 GHz. The parallel algorithm was implemented in ANSI C programming language and MPI was used for parallelization. Runs were performed with different combinations of parameters on the cluster (160 IBM PowerPC-2200 processors) in the Ioffe Physical Technical Institute of the Russian Academy of Sciences, St.Petersburg, on the cluster (1980 Intel Xeons) in the Joint Supercomputer Center of the Russian

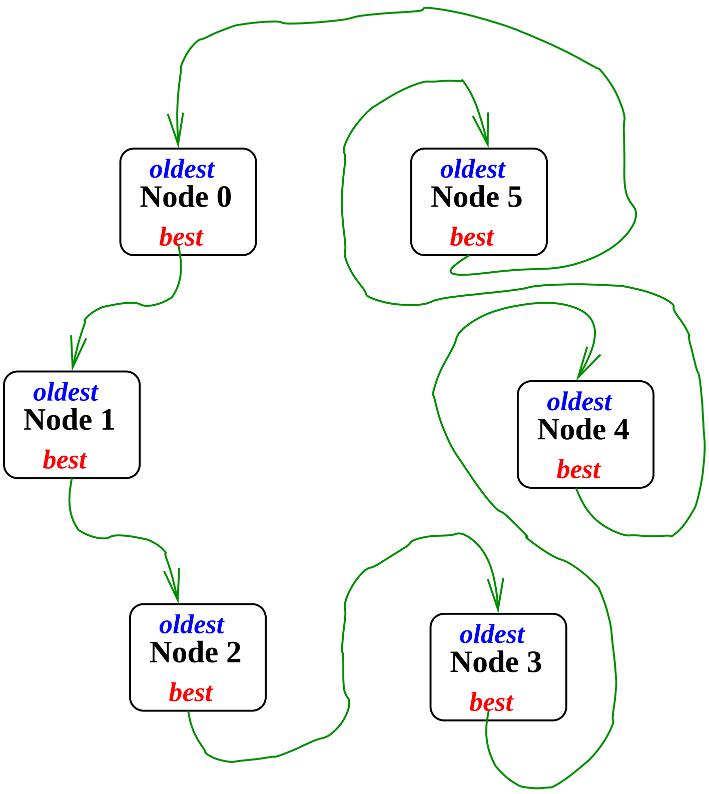

Figure 4: Migration scheme. Network topology

Academy of Sciences, Moscow, and on the cluster (128 AMD Opterons 280) in the Laboratory of Applied Mathematics and Mechanics of the St.Petersburg State Polytechnical University.

1.8 Parameters of algorithm

The following parameters were used:

- Population size:268;
- Control parameters γ:0.95;
- Stopping criterion M:50 and ρ:1e-2;

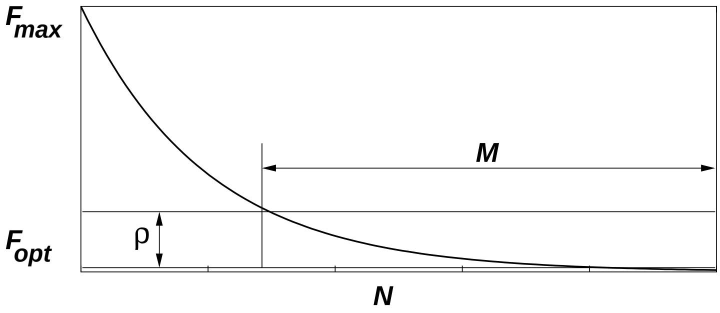

Figure 5: Stopping criterion. The value of quality functional F is plotted against the number of iterations N

- Transformation parameter η:0.05;
- Each 2 iterations 5 oldest members of $k+1$ node were substituted with 5 best members of k node;
- Number of branches:5.

The seed for random number generator was different for each run.

References

[1] K. N. Lurie. *Applied optimal control theory of distributed systems.* Plenum Press, New York, NY, U.S.A., 1993. 1

[2] Hui-Yuan Fan and Jouni Lampinen. A trigonometric mutation operation to differential evolution. *Journal of Global Optimization*, 27:105–129, 2003. 2

[3] Koumoutsakos P. Gaemperle R., Mueller S. D. A Parameter Study for Differential Evolution. In N. E. Mastorakis A. Grmela, editor, *Advances in Intelligent Systems, Fuzzy Systems, Evolutionary Computation*, pages 293–298. WSEAS Press, 2002. 3

[4] Zaharie D. Parameter adaptation in differential evolution by controlling the population diversity. In D.Petcu, editor, *Proc. of 4th International-Workshop on Symbolic and Numeric Algorithms for Scientific Computing*, pages 385–397, Timisoara, Romania, 2002. 3

[5] Tasoulis D.K., Pavlidis N.G., Plagianakos V.P., and Vrahatis. M.N. Parallel differential evolution. In *In Congress on Evolutionary Computation (CEC 2004)*, Portland, Oregon, 2004. 3

[6] Konstantin Kozlov and Alexander Samsonov. DEEP – Differential Evolution Entirely Parallel Method for Gene Regulatory Networks. *Journal of Supercomputing*, 2010. 4
