# Supplementary material for: Modeling of Gap Gene Expression in Drosophila Kruppel Mutants
Source: PLoS Comput Biol. 2012 Aug 23;8(8):e1002635. doi: 10.1371/journal.pcbi.1002635 (PMC3426564; doi:10.1371/journal.pcbi.1002635)
Supplement: Text S2 — Supplementary methods. Identifiability analysis. (PDF) [file pcbi.1002635.s010.pdf]

Identifiability analysis

For the comprehensive analysis of modeling results it is necessary to know how reliable the parameter estimates are. In practice insufficient or noisy data, as well as the strong parameter correlation or even their functional relation may prevent the unambiguous determination of parameter values. In addition, many models used in systems biology exhibit parameter "sloppiness" [1]. This means that there may exist model parameters, estimations of which can vary by orders of magnitude without significantly influencing the quality of fit. These uncertainties may become particularly problematic when models are used explicitly to extract biological information from estimated parameter values or for prediction of dynamical behavior of the model at different parameter values. For example, if several parameter values are fixed, and if these parameters are correlated with those estimated by fitting, the correct prediction of the system behavior may become infeasible. The detection of non-identifiable and sloppy parameters is the subject of identifiability analysis.

Basically two approaches are used to handle non-identifiability: first, the model structure itself is investigated with respect to non-identifiabilities. If such non-identifiabilities exist, they must be removed analytically by introduction of new parameters, representing, e.g. an identifiable combination of several non-identifiable parameters. This approach is referred to as *a priori* or structural identifiability analysis, as the model structure is examined before simulating and fitting procedures. Within the second approach, *a posteriori* or practical identifiability study, non-identifiabilities are detected by fitting to data and investigating parameter estimates. Besides, parameter identifiability can be addressed either locally near a given point or globally over the whole parameter space. In the current study we apply two local *a posteriori* approaches to verify the reliability of parameter estimates, their correlations and to determine non-identifiable and sloppy parameters.

Method based on asymptotic confidence intervals

The first method is based on asymptotic confidence intervals [2, 3]. This method is the most commonly used approach to the local identifiability analysis. Let $\widehat{\theta}$ be a parameter vector of size m which minimizes

$$S(\theta) = \sum_{i=1}^{N} (y_i(t_i, \theta) - \widetilde{y}_i)^2 = Y^T(\theta)Y(\theta),$$

where $\widetilde{y}_i$ is an observed value, $y_i(t_i, \theta)$ is the corresponding model value, N is a number of observations. If measurement errors are independent and normally distributed, $\widehat{\theta}_i$ are the maximum likelihood estimates. Then the asymptotic (1- α)-confidence region for the 'true' parameter vector θ is determined from the inequality

$$(\theta - \widehat{\theta})^T \left(J^T J\right) (\theta - \widehat{\theta}) \leq \frac{m}{N-m} S(\theta) F_{\alpha,m,N-m}, \tag{1}$$

where the Jacobian $J = J(\theta) = \partial Y(\theta)/\partial\theta$ is the so-called sensitivity matrix of size $N \times m$; $F_{\alpha,m,N-m}$ is an α-quantile of F-distribution with m and $N-m$ degrees of freedom. The inverse of the matrix $J^T(\theta)J(\theta)$ multiplied by the variance of observation error is the covariance matrix of the parameter estimates.

The confidence intervals for an individual parameter θ_i in case of independent parameters can be expressed as

$$(\theta_i - \widehat{\theta}_i)^2 \leq \frac{m}{N-m} S(\theta) F_{\alpha,m,N-m} (J^T J)^{-1}_{ii}. \tag{2}$$

The size of confidence intervals characterizes the sensitivity of the model solution to parameter changes and hence the reliability of the parameter estimate. If the search space is limited, confidence intervals exceeding the parameter limits indicate the parameter sloppiness and hence non-identifiability. However, if some parameters are strongly correlated the confidence intervals (2) which represent the projection of the confidence area (1) onto the ith parameter axis are overestimated. In other words the confidence

interval (2) is the whole area of the parameter variation as the other parameters take any possible values from the m-dimensional area (1). In case of parameter correlation the confidence region is asymmetric and its principal axes are inclined with respect to the parameter axes. As a result its projection, confidence interval, is much larger than any intersection of the region with any line parallel to parameter axis. Such an intersection represents confidence interval computed for the parameter as all the other parameter values are fixed within the ellipsoid (1). This approach is illustrated in Figure 1 for two-parameter case.

High off-diagonal elements of the covariance matrix $(J^T J)^{-1}$ indicate strong linear dependencies of parameter estimates. With respect to parameter identifiability this means that the effects of changes in one parameter values on the model output can be compensated by the changes in other parameters. In other words, different parameter values can lead to nearly the same model output; that is, the involved parameters are poorly identifiable. However, the correlation matrix does not provide exhaustive information about parameter identifiability in a high-dimensional parametric model, as it only reveals the pairwise parameter correlations not being able to detect relations of higher order between three and more parameters. In case of strong linear relations between parameters the matrix $J^T J$ is ill-conditioned and its inversion is infeasible. A standard approximation of the inverse of an ill-conditioned matrix M is the Moore-Penrose pseudoinverse M^+ that is computed as $(M^T M + \varepsilon I)^{-1} M^T$, where ε is a small positive number.

However, due to numerical difficulties the size of confidence intervals is very sensitive to the choice of ε. To verify the identifiability results we apply the method exploiting another type of confidence intervals based on profile likelihood [4]. The accuracy of pseudo-inversion algorithm thus is chosen so that two types of confidence intervals provide the same set of identifiable parameters. The profile likelihood (PL) is the likelihood function minimized with respect to all the parameters except one parameter which is fixed

$$S_{PL,j}(\theta_j^*) = \min_{\{\theta_i : i \neq j\}} S(\theta)|_{\theta_j = \theta_j^*}. \tag{3}$$

The likelihood-based $(1-\alpha)$-confidence interval for θ_j is defined by

$$\{\theta_j : \frac{S_{PL}(\theta_j) - S(\widehat{\theta})}{S(\widehat{\theta})} \leq \frac{1}{N-m} F_{\alpha,1,N-m}\}. \tag{4}$$

These confidence intervals are known to be more accurate than the asymptotic ones for finite samples, moreover the method doesn't require an inversion of ill-conditioned matrix and allows to avoid computational errors.

To make sure that the estimate of parameter θ_j insignificantly differs from zero we compute the value of $S_{PL,j}(0)$ and check whether $\theta_j = 0$ satisfies (4).

Collinearity analysis of the sensitivity matrix

The other method to detect interrelations between parameters is the collinearity analysis presented in [5]. The method is suitable for models with large number of parameters. The aim of the method is to reveal the so-called near collinear columns of the sensitivity matrix $J(\theta) = \partial Y(\theta)/\partial\theta$, and thus detect the subsets of identifiable and non-identifiable parameters.

Columns of a matrix A are called *near collinear* if there exists a vector $\beta = (\beta_1, ..., \beta_m)^T$ such that $\|\beta\| \neq 0$ and $A\beta \approx 0$. We consider all subsets, K, of k parameters ($k \leq m$) from the whole set of m parameters and the corresponding submatrices $\widetilde{J}_K$ of size $N \times k$ of the normalized sensitivity matrix. The matrix $\widetilde{J}_K$ contains the columns $\frac{J_j}{\|J_j\|}$, where J_j is a jth column of the matrix J; indices j belong to the subset K. The *collinearity index* of $\widetilde{J}_K$ is defined as

$$\gamma_k = \frac{1}{\min_{\|\beta\|=1} \|\widetilde{J}_K \beta\|} = \frac{1}{\sqrt{\lambda_k}}, \tag{5}$$

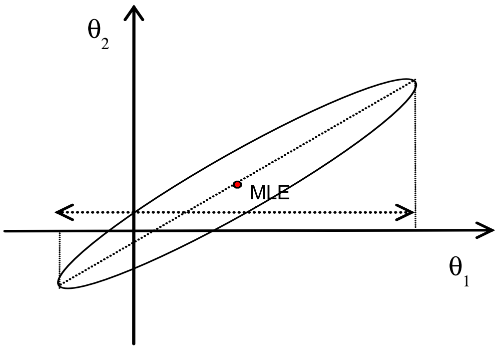

Figure 1. Confidence area in case of two-dimensional parameter space. Maximum likelihood estimate of the 2D parameter is labeled by a red spot. The asymmetric shape and inclination of the confidence area indicates the parameter dependency. Projection of the 2D area onto the θ_1 axis constitutes the confidence interval for this parameter (marked by a double-sided arrow).

where λ_k is the minimal eigenvalue of the matrix $\widetilde{J}_K^T \widetilde{J}_K$.

The collinearity index has a simple interpretation: A change in the output vector $Y(\theta)$ caused by a shift of a parameter $\theta_j \in K$ can be compensated in the linear approximation up to a fraction $1/\gamma_k$ by appropriate changes in the other parameters in K. High values of γ_k indicate that the subset of parameters K is poorly identifiable due to relations between at least two parameters.

The collinearity index is computed for all the subsets of the parameter space of all dimensions $k < m$. The aim of the analysis is to detect all the parameter subsets with high collinearity index such that they do not contain subsets of lower dimension for which the collinearity index is also high. Thus we reveal all the non-identifiable parameters. The subsets with low collinearity index are identifiable.

References

1. Gutenkunst R, Waterfall J, Casey F, Brown K, Myers C, et al. (2007) Universally sloppy parameter sensitivities in systems biology models. PLoS computational biology 3: 1871–78.

2. Bates DM, Watts DG (1988) Nonlinear Regression Analysis and its Applications. John Wiley.

3. Ashyraliyev M, Jaeger J, Blom JG (2008) Parameter estimation and determinability analysis applied to drosophila gap gene circuits. BMC Syst Biol 2: 83.

4. Raue A, Kreutz C, Maiwald T, Bachmann J, Schilling M, et al. (2009) Structural and practical identifiability analysis of partially observed dynamical models by exploiting the profile likelihood. Bioinformatics 25: 1923–1929.

5. Brun R, Reichert P, Kunsch H (2001) Practical identifiability analysis of large environmental simulation models. Water Resources Research 37: 1015–1030.
